# Determining the minimum number of protein-protein interactions required to support known protein complexes

**DOI:** 10.1371/journal.pone.0195545

**Published:** 2018-04-26

**Authors:** Natsu Nakajima, Morihiro Hayashida, Jesper Jansson, Osamu Maruyama, Tatsuya Akutsu

**Affiliations:** 1 Institute of Molecular and Cellular Biosciences, The University of Tokyo, 1-1-1, Yayoi, Bunkyo-ku, Tokyo 113-0032, Japan; 2 Department of Electrical Engineering and Computer Science, National Institute of Technology, Matsue College, 14-4, Nishiikumacho, Matsue, Shimane 690-8518, Japan; 3 Department of Computing, The Hong Kong Polytechnic University, Hung Hom, Kowloon, Hong Kong; 4 Institute of Mathematics for Industry, Kyushu University, 744 Motooka, Nishi-ku, Fukuoka 819-0395, Japan; 5 Bioinformatics Center, Institute for Chemical Research, Kyoto University, Gokasho, Uji, Kyoto 611-0011, Japan; Texas A&M University College Station, UNITED STATES

## Abstract

The prediction of protein complexes from protein-protein interactions (PPIs) is a well-studied problem in bioinformatics. However, the currently available PPI data is not enough to describe all known protein complexes. In this paper, we express the problem of determining the minimum number of (additional) required protein-protein interactions as a graph theoretic problem under the constraint that each complex constitutes a connected component in a PPI network. For this problem, we develop two computational methods: one is based on integer linear programming (ILPMinPPI) and the other one is based on an existing greedy-type approximation algorithm (GreedyMinPPI) originally developed in the context of communication and social networks. Since the former method is only applicable to datasets of small size, we apply the latter method to a combination of the CYC2008 protein complex dataset and each of eight PPI datasets (STRING, MINT, BioGRID, IntAct, DIP, BIND, WI-PHI, iRefIndex). The results show that the minimum number of additional required PPIs ranges from 51 (STRING) to 964 (BIND), and that even the four best PPI databases, STRING (51), BioGRID (67), WI-PHI (93) and iRefIndex (85), do not include enough PPIs to form all CYC2008 protein complexes. We also demonstrate that the proposed problem framework and our solutions can enhance the prediction accuracy of existing PPI prediction methods. ILPMinPPI can be freely downloaded from http://sunflower.kuicr.kyoto-u.ac.jp/~nakajima/.

## Introduction

Identification of protein complexes is important for understanding cellular mechanisms because many proteins express their functions by forming complexes. Since it is difficult to experimentally determine protein complexes, extensive studies have been done on the prediction of protein complexes. Among them, many studies focused on the effective use of protein-protein interaction (PPI) data because proteins in a complex physically interact and a large amount of PPI data has been available due to developments of high-throughput experimental techniques [1, 2]. In most of these studies, protein complexes are predicted by identifying non-overlapping or overlapping clusters (i.e., certain types of connected subgraphs) in PPI networks possibly with other biological information. In order to identify clusters in PPI networks, various methods have been developed, including the Markov CLuster (MCL) method [3], the Molecular Complex Detection (MCODE) method [4], the Restricted Neighbourhood Search Clustering (RNSC) method [5], the Repeated Random Walks (RRW) method [6], the Clustering based on Maximal Clique (CMC) method [7], and the Node-Weighted Expansion (NWE) method [8]. However, it was also pointed out that known PPI data suffers from a significant amount of noise in terms of both false positives (spuriously detected interactions) and false negatives (missing interactions) [9]. Therefore, various methods have also been developed that make use of weight and/or reliability of PPIs [7, 9, 10].

When studying and analyzing these protein complex prediction methods, we encounter a fundamental question. Is the current PPI data enough to explain all known protein complexes? If not, how many additional PPIs are required? The main purpose of this paper is to tackle this fundamental question. In order to answer the question, we impose as a minimum requirement that the subgraph induced by the nodes (i.e., proteins) in each complex must be connected. The answer would enable us to deduce that many interactions to detect the associations between proteins forming all known protein complexes are missing from the current PPI data and if the number of the additional PPIs is large, more additional experiments might be necessary. Then, we define the problem of determining the minimum number of additional PPIs required to support known complexes (MinPPI) as: given a set of protein complexes (i.e., a set of sets of proteins) and a set of PPIs (i.e., a set of edges among proteins), find a minimum number of additional PPIs such that the connectivity requirement is satisfied for all given protein complexes. We also define MinPPI0 as the special case of MinPPI in which the set of given PPIs is empty.

Interestingly, the same problem has been studied in the analysis of communication and social networks under the name of the Network Construction problem and a greedy, polynomial-time approximation algorithm for it has been proposed [11]. This fact suggests that our question is a natural and general one. We modify this greedy-type algorithm so that we can start with some known PPI data, and the resulting algorithm is called GreedyMinPPI. We also develop a novel integer linear programming (ILP)-based method called ILPMinPPI that gives an exact solution.

In this paper, we compare two methods using moderate size synthetic data. Then, we apply GreedyMinPPI to three large-scale real protein complex datasets, CYC2008 [12], MIPS [13], and Aloy *et al*.’s set [1, 14] without any known PPIs, to estimate the minimum number of PPIs, and to pairs of CYC2008 and eight PPI datasets (STRING [15], MINT [16], BioGRID [17], DIP [18], BIND [19], WI-PHI [20], IntAct [21, 22], and iRefIndex [23]) to estimate the minimum number of additional PPIs.

However, as mentioned above, known PPI data suffers from a significant amount of noise. In particular, there are a large amount of missing interactions [24, 25]. Therefore, many methods have been proposed to predict PPIs from protein sequences, protein structures, and/or other biological data [26–30]. Since GreedyMinPPI outputs also unknown PPIs, it might be helpful to enhance existing PPI prediction methods by GreedyMinPPI. In order to assess the usefulness of this idea, we examine a combination of GreedyMinPPI and each of four state-of-the-art prediction methods for weighted PPIs, Struct2Net [26], ENTS [27], PIP [28], and iWRAP [29], using four PPI datasets extracted from STRING [15], MINT [16], WI-PHI [20], and IntAct [21]. Since the four databases contain interactions with confidence score based on distinct sources of evidence, we regard these PPI databases as reliable.

## Problem definition and motivation

The two problems *gMinPPI* and *gMinPPI0* studied in this paper are defined formally as graph theoretic problems as follows. The input to gMinPPI is an undirected graph *G* = (*V*, *E*), where *V* is a set of vertices and *E* is a set of edges, along with a collection C of subsets of *V*, and the output is an undirected graph *G*′ = (*V*, *E* ∪ *E*′), where *E*′ is a set of additional edges, such that the subgraph of *G*′ induced by each $C_{i}\in C$ is connected and the value of |*E*′| is minimized. gMinPPI0 is the special case of gMinPPI where *E* = ∅. Given any instance of gMinPPI or gMinPPI0, we use the notation *n* = |*V*| and m=|C|. When we apply gMinPPI and gMinPPI0 to the protein complex data, which are also defined as MinPPI and MinPPI0, which means that MinPPI0 is the special case of MinPPI where the set of given PPIs is empty.

**Fig 1:**
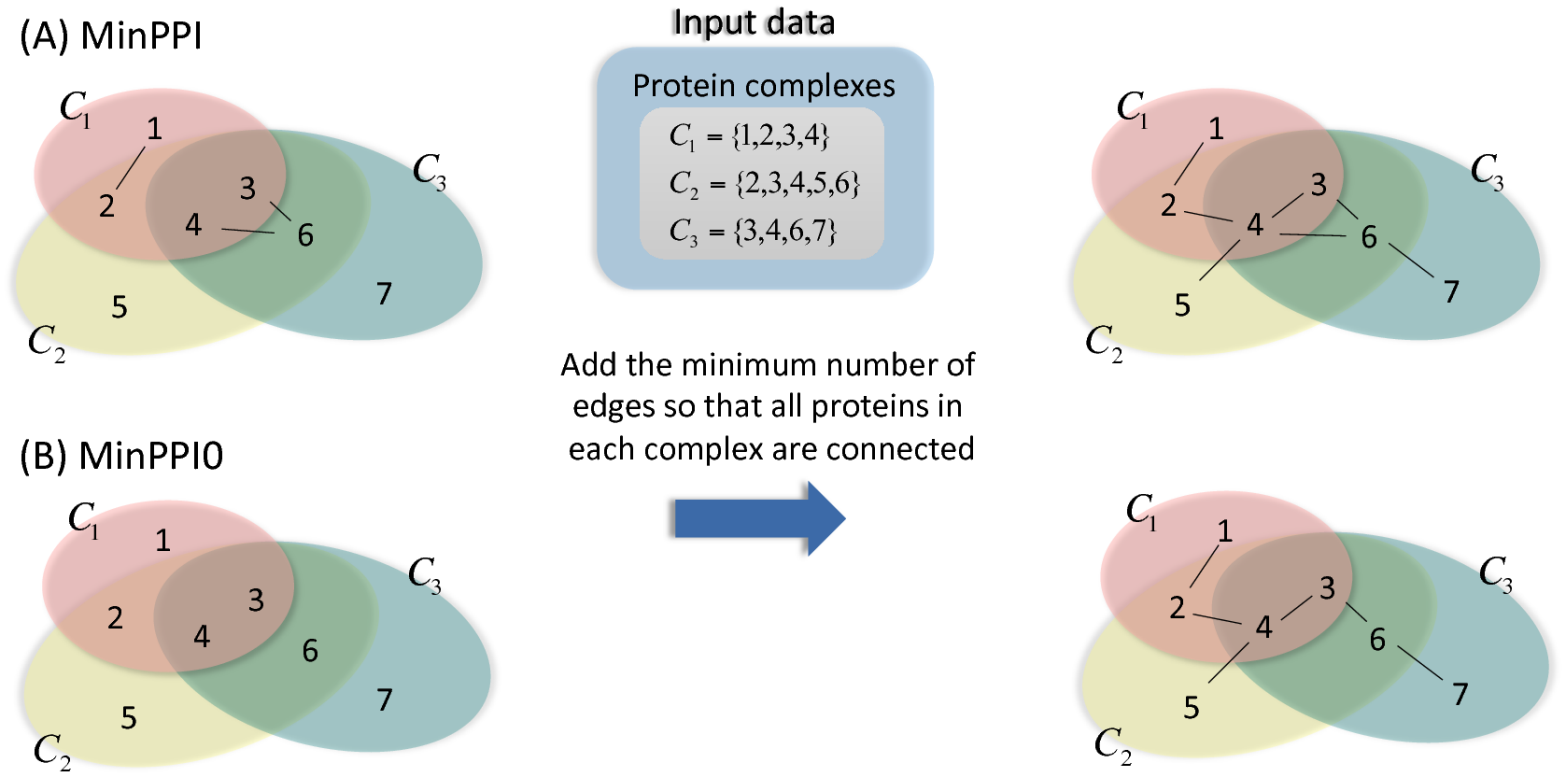
A description of MinPPI and MinPPI0. (A) An example of MinPPI. MinPPI corresponds to determining the minimum number of additional interactions starting with known PPI (a set of edges among proteins) data. For example, if a family of protein complexes C consisting of *C*_1_, *C*_2_ and *C*_3_ where the total number of proteins is 7 and known PPI data are given as an input data, the objective is to find the minimum number of additional PPIs required to describe interactions in all given protein complexes such that all proteins belonging to each complex *C*_*i*_ (*i* = 1, 2, 3) must be connected. Since an initial PPI network has 3 edges and one protein complex overlaps with other protein complexes, the resulting graph contains 7 edges. (B) An example of MinPPI0. MinPPI0 corresponds to the determination beginning with the set of known PPIs is empty. For example, if a family of protein complexes C composed of *C*_1_, *C*_2_ and *C*_3_ and known PPI data with no edge are given as an input data, the objective is to find the connected graph which has the minimum number of PPIs such that all proteins belonging to each complex *C*_*i*_ (*i* = 1, 2, 3) must be connected. Since a given PPI network has no edges and one protein complex overlaps with others, the number of additional edges is 6.

In our application, the elements in *V*, *E*, and C represent proteins, known protein-protein interactions (PPIs), and protein complexes, respectively. The elements in *E*′ correspond to hypothetical PPIs whose existence would guarantee that each protein complex is internally connected. See Fig 1 for two examples. Hence the value of |*E*′| is a lower bound on the number of additional PPIs needed to support the given protein complexes. The motivation of this study is to solve MinPPI for some particular data sets from the literature and investigate their values of |*E*′|; if |*E*′| for some data set is large, this suggests that many interactions between proteins are missing from the database and that additional experiments might be necessary to complete the picture.

## Previous work

In the literature, gMinPPI0 is equivalent to the *Minimum Topic-Connected Overlay* problem [31] and the *Uniform Cost Network Construction* problem [11]. The more general *Network Construction* problem introduced in [11] is an extension of gMinPPI0 in which a non-negative cost of an edge *c*_*e*_ for each *e* ∈ *V* × *V* is allowed and aims to minimize ∑_*e*∈*E*′_ *c*_*e*_. Restricted versions of the Network Construction problem in which the output graph *G*′ is required to be a tree or a star were studied in [32] and [33] (note that for certain inputs, no such *G*′ exists). Chockler *et al*. [31] proved that gMinPPI0 is NP-hard to approximate within a constant factor. They also gave a polynomial-time, greedy approximation algorithm for gMinPPI0 which starts with *E*′ = ∅ and inserts one suitably chosen edge at a time into *E*′ until each $C_{i}\in C$ induces a single connected component in the resulting graph *G*′, and showed that its approximation ratio is logarithmic in $\sum_{C_{i}\in C}|C_{i}|$. Angluin *et al*. [11] recently strengthened these results by: (i) proving that the Network Construction problem and equivalently, gMinPPI0 is NP-hard to approximate within a factor of Ω(log *n*); and (ii) extending the greedy approximation algorithm for gMinPPI0 and refining its mathematical analysis to obtain a polynomial-time *O*(log *m*)-approximation algorithm for the Network Construction problem.

## Materials and methods

### Integer linear programming formulation

We propose an exact method called ILPMinPPI, for the problem of predicting PPIs beginning with the set of known PPIs is empty (corresponding MinPPI0).

MinPPI0 can be formulated using the following integer linear programming (ILP). For each complex *C*_*p*_, we add the following constraints using different 0-1 variables for different *C*_*p*_,

$$\begin{array}{c} \begin{array}{cc} x_{ij}^{p,T_{p}} & =1\;\;\text{for}\;\text{all}\;i<j,\\ x_{ij}^{p,0} & \leq e_{ij}\;\;\text{for}\;\text{all}\;i<j,\\ x_{ij}^{p,t+1} & \leq x_{ij}^{p,t}+\sum_{k\notin \{i,j\}}x_{ij,k}^{p,t+1}\;\;\text{for}\;\text{all}\;i<j\;\text{and}\;k\notin \left\{i,j\right\},\\ x_{ij,k}^{p,t+1} & \leq \frac{1}{2}(x_{ik}^{p,t}+x_{kj}^{p,t})\;\;\text{for}\;\text{all}\;i<j\;\text{and}\;k\notin \left\{i,j\right\}, \end{array} \end{array}$$ (1)

where *i*, *j*, *k* ∈ *C*_*p*_, $x_{ij}^{p,t}=x_{ji}^{p,t}$, $x_{ij,k}^{p,t}=x_{ji,k}^{p,t}$, and *T*_*p*_ = ⌈log|*C*_*p*_|⌉. *e*_*ij*_ is a variable which indicates whether an edge exists between proteins *i* and *j*. If *e*_*ij*_ = 1, an interaction exists between proteins *i* and *j*. $x_{ij}^{p,t}$ reflects whether proteins *i* and *j* are connected after *t* (0≤*t*≤*T*_*p*_) steps and $x_{ij,k}^{p,t}$ reflects whether proteins *i* and *j* are connected through protein *k* after *t* steps. The third constraint means that if *i* and *k* are connected and *k* and *j* are connected, *i* and *j* are also connected. Hence, after enough steps *T*_*p*_, proteins *i* and *j* should be connected, which means that $x_{ij}^{p,T_{p}}$ takes 1. Since the connectedness of each complex is checked by using the doubling technique, it is enough to repeat this process at most *O*(log|*C*_*p*_|) steps. Hence, these constraints state that the subgraph of *G*(*V*, *E*) induced by nodes (proteins) in each *C*_*p*_ must be connected, which guarantees that each protein complex is internally connected under the condition that edges with *e*_*ij*_ = 1 can only be used. Then, the required ILP is formulated as

$$\begin{array}{c} \text{minimize}\;\sum_{1\leq i<j\leq n}e_{ij}, \end{array}$$ (2)

with constraints for all *C*_*p*_s.

### Approximation algorithm

The exact method ILPMinPPI requires exponential time and can thus only be applied to small datasets in practice. To deal with large-scale datasets, we now extend the approximation algorithm of [11] to MinPPI. The resulting method will be referred to as GreedyMinPPI. It enables us to detect the interactions among proteins with most highly overlapping and most of these interactions would be expected to be more reliable. This means that in GreedyMinPPI, the reliability or confidence score of PPIs are assigned in the order in which interactions are detected.

#### An *O*(log *m*)-approximation algorithm for gMinPPI

Angluin *et al*. [11] proved that the Network Construction problem can be approximated within a ratio of *O*(log *m*) in polynomial time. We now show that this yields a polynomial-time, *O*(log *m*)-approximation for gMinPPI.

**Theorem 1**. *gMinPPI can be approximated within a ratio of O*(log *m*) *in polynomial time*.

*Proof*. Let (G;C) be any given instance of gMinPPI, where *G* = (*V*, *E*). Create an instance (V;C;c) of the Network Construction problem by defining a cost function *c* on pairs of vertices as follows: for every *u*, *v* ∈ *V* with *u* ≠ *v*, if {*u*, *v*} ∉ *E* then *c*({*u*, *v*}) = 1 and if {*u*, *v*} ∈ *E* then *c*({*u*, *v*}) = *ϵ*, where *ϵ* is any constant satisfying 0 < *ϵ* < 2^−*n*^ (i.e., *n* = |*V*|). Next, apply Angluin *et al*.’s *O*(log *m*)-approximation algorithm [11] to (V;C;c), and denote the obtained graph by (*V*, E*). Finally, output the graph (*V*, *E* ∪ E*) as the approximate solution to gMinPPI.

Clearly, the running time is polynomial. To bound the approximation ratio, let *OPT* be any optimal gMinPPI-solution to the given (G;C). Denote the total number of edges in *OPT* by |*E*| + *x*. Note that (G;C) admits a solution to gMinPPI with |*E*| + *x* edges if and only if (V;C;c) admits a solution to the Network Construction problem whose cost (i.e., sum of costs of all edges) is in the interval [*x*, *x* + *ϵ* ⋅ |*E*|]. Since (*V*, E*) is an *O*(log *m*)-approximate solution for the latter, the output (*V*, *E* ∪ E*) contains at most |*E*| + (*x* + 1) ⋅ *O*(log *m*) = |*E*| + *x* ⋅ *O*(log *m*) edges and is therefore an *O*(log *m*)-approximation of *OPT*.

## Results

### Computer environment

We evaluated the performance of both ILPMinPPI and GreedyMinPPI using both synthetic data and real protein-protein interaction data. An integer programming solver, CPLEX Interactive Optimizer 12.4.0.0 (http://www-01.ibm.com/software/commerce/optimization/cplex-optimizer/) was used to compute an exact optimal solution and ‘Epi’ library (version 1.1.67) in R (version 3.2.1) was used to plot the ROC curve. The implementation of ILPMinPPI and GreedyMinPPI was done by C/C++ code. The C/C++ programs are used to generate linear programs which are then fed to CPLEX. All experiments were performed on a PC with Intel Core i7-2600 CPU (3.40 GHz) with 7.7 GB RAM running under the Fedora 21 with Linux kernel 3.14.2 and with Xeon E5-2667 CPU (3.30GHz×8) with 62.9 GiB memory running under the Mint 17.1 Cinnamon with Linux kernel 3.13.0-37-generic. We used the same CPU to compare the CPU time in each experiment. ILPMinPPI can be freely downloaded from http://sunflower.kuicr.kyoto-u.ac.jp/~nakajima/.

### Results on ILPMinPPI using synthetic data and real data

#### Comparison of ILPMinPPI and GreedyMinPPI using synthetic data

**Fig 2:**
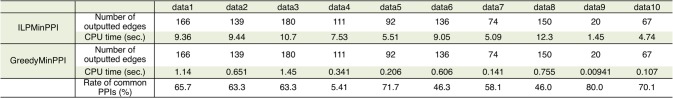
Performance evaluation of ILPMinPPI and GreedyMinPPI using synthetic data (syndata 2).

At the beginning, we compared GreedyMinPPI with ILPMinPPI using two types of synthetic datasets. We randomly built two datasets (syndata 1, syndata 2), each of which is composed of 10 artificial protein complex datasets (data1–data10), where the maximum number of total proteins, complexes and proteins within a complex are 10, 20 and 5 for syndata 1, and both 100 and 4 for syndata 2, respectively. Furthermore, in real world datasets, since the proteins apparently outnumbered the protein complexes, we also performed the simulation experiments in three types of practical setting (see details in Table A1(a) in S1 File). For each protein complex dataset, the corresponding ILP formulation is written in the LP-format required by CPLEX. Then, we evaluated and compared the performance of ILPMinPPI with that of GreedyMinPPI by measuring the total number of interactions (edges) and the CPU time (real time) as shown in Fig 2 and Table A1 in S1 File.

For example, the results indicate that ILPMinPPI outputted 166 edges requiring 9.36 sec using data1 of syndata 2 shown in Fig 2. For syndata 1, since the objective values provided by the two methods are not all the same, we count the number of common PPIs. Table A1(b) in S1 File shows that two methods do not always output the same objective values but the optimal values are relatively close. On the other hand, both ILPMinPPI and GreedyMinPPI provide the same objective values in all cases of syndata 2, 3 and 4, however, the rate of the common PPIs between the two methods is not so high.

Although ILPMinPPI outputs the same objective value for the same complex datasets, this property is not guaranteed for GreedyMinPPI because the objective value may depend on the ordering of the input data. In order to validate whether or not GreedyMinPPI outputs the same prediction results among runs with the same input data but different orderings, we also examined GreedyMinPPI on MinPPI0 using randomly generated datasets, where the maximum numbers of proteins and complexes were 1600 and 400, respectively, and the maximum number of proteins within one complex was 5. The configuration of each dataset was changed by shuffling the complexes and the subunits each 10 times. Therefore, 100 configurations were examined for each dataset. The results are summarized in Table A2(a) in S1 File. We found that all approximate solutions were exactly the same including the addition order of edges. It is reasonable because GreedyMinPPI repeatedly detects the protein pair with the highest overlap by using the confidence score and thus it is not plausible that multiple pairs have the same confidence score. However, there existed one exceptional case when GreedyMinPPI was applied to real protein complex datasets (see Table A2(b) in S1 File). For the STRING dataset, 51 protein pairs were identified in 91 trials whereas 139 protein pairs were identified in 9 trials. However, the number of bad cases (i.e., 139 protein pairs) is small. Therefore, it is expected that even in non-preferred cases, we can obtain a reasonably good solution by examining multiple configurations and taking the best solution.

Additionally, the CPU time of GreedyMinPPI is much less than that of ILPMinPPI only if the dataset is small. It must be noted that ILPMinPPI and GreedyMinPPI could possibly work when one protein complex consists of relatively few proteins, because the CPU time depends on the maximum number of proteins as described in Table A3 in S1 File. Actually, ILPMinPPI could not work on syndata 5. GreedyMinPPI provides optimal or near-optimal solutions while reducing the CPU time and it might therefore be effective for large datasets. This result also suggests that ILPMinPPI still has room for improvement by extending the ILP formulation to avoid combinatorial explosion.

#### Results using protein complex dataset

Although ILPMinPPI is not efficient for large data in general, it would be insightful to examine whether it can provide optimal solutions for real datasets. We thus applied ILPMinPPI to MinPPI0 using CYC2008 protein complexes as a benchmark set reported by Pu *et al*. [12]. CYC2008 consists of 408 protein complexes, some of which are composed of dozens of distinct subunits shared between the different complexes.

**Fig 3:**
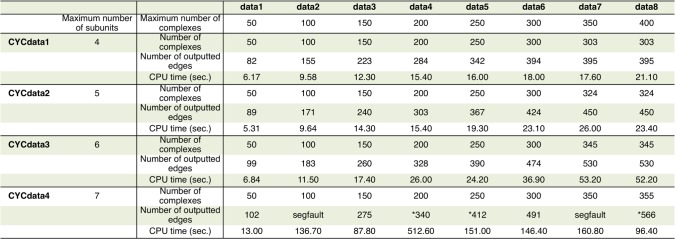
Performance comparison of ILPMinPPI with four protein complex datasets. Summary of four real protein complex datasets from CYC2008 and performance comparison of ILPMinPPI with these datasets. For example, ‘data7’ of CYCdata1 is composed of 303 complexes (see ‘Number of complexes’), where the number of subunits is at most 4 (see ‘Maximum number of subunits’). When using this data, ILPMinPPI outputs 395 edges and requires 17.6 seconds. A ‘segfault’ refers to the segmentation fault which occurs when a program accesses an invalid memory address and the number of outputted edges with asterisk(*) means that CPLEX outputs not an optimal solution but a feasible solution because of exceeding the memory limit.

To examine how the CPU time is affected by the size of a dataset and the number of subunits, we prepare four datasets derived from the benchmark complexes according to the number of proteins within a complex. Unfortunately, the results on synthetic data indicate that ILPMinPPI can be applicable for the limited datasets including complexes composed of only a few subunits. Therefore, we randomly constructed the datasets that are limited to the maximum number of proteins within a complex as ranging from 4 to 10 and then examine how the performance of ILPMinPPI evolves as the maximum number of complexes increases ranging from 50 to 400 with 50 intervals in order from the beginning of CYC2008. For example, as shown in Fig 3, CYCdata1 consists of 8 components (data1–data8), each of which is composed of a large set of protein complexes formed by at most 4 proteins.

Fig 3 indicates that there may be some correlation between the CPU time and the maximum number of subunits. If a complex is formed by at most 6 subunits, ILPMinPPI requires much less or slowly increasing CPU time, regardless of the size of the complex. Compared with the performance on synthetic data, almost the same CPU time is required for this method when using the syndata 2 shown in Table A1(c) in S1 File. In contrast, in case that the number of subunits is more than 7, it cannot provide an optimal solution by solving the MIP problem on CPLEX due to the high memory consumption. Indeed, MinPPI0 on these datasets almost always lead to exponential increase in CPU time or segmentation faults, some of which are not listed in Fig 3 if the complex is composed of 8–10 subunits. Therefore, these results reveal that the computational complexity of ILPMinPPI increases exponentially because of depending not on the total number of complexes but on the maximum number of subunits.

In this experiment, an optimal solution can be provided within a reasonable CPU time only if the relatively small number of subunits are included in the complex, note however that, in reality, there also even exist several complexes formed by more than 10 subunits in CYC2008 and more than 30 subunits in MIPS [13] as summarized in Table A3(b) in S1 File. The current ILPMinPPI has room for improvement to reduce the computational costs. One possibility is that there needs to be a reduction of the size of the MIP problem by omitting the unnecessary constraints or to increase the maximum memory usage.

### Results on GreedyMinPPI using real data

#### The number of PPI for three protein complex datasets

We only tested GreedyMinPPI to solve MinPPI0 using real large-scale protein complex datasets because the current ILPMinPPI is not suitable for application to large-scale datasets (it is only applicable to datasets of small size consisting of 100-150 nodes). We used a dataset derived from CYC2008 [12] which consists of 408 protein complexes involving 1627 proteins in *S. cerevisiae*, and also used two protein complex datasets obtained from MIPS [13] which was publicly available from the study of [34] and Aloy *et al*. [1, 14].

**Fig 4:**
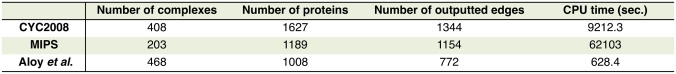
Results with three protein complex datasets.

We applied GreedyMinPPI to three protein complex datasets and evaluated the performance in terms of the total number of outputted PPIs and the execution time (CPU time). To test the correlation between the CPU time and the protein complex formation, we counted the number of complexes consisting of *p*_*n*_ proteins. The results are presented in Fig 4 and Table A3 in S1 File. It is observed that the number of added edges is less than the number of proteins in all cases. It is reasonable because three protein complex datasets may consist of the disjoint sets of protein complexes which are a set of unions of proteins (overlapping proteins). For example, MIPS dataset is composed of a small number of disjoint sets of complexes in which a lot of proteins overlap and this may be related to the increased CPU usage as described below. In contrast, Aloy *et al*.’s dataset consists of a large number of disjoint sets of complexes that are a small number of overlapping complexes, despite containing lots of complexes. Similarly, as you can see in Fig 4 and Table A3(b) in S1 File, the increase on required CPU time may also be caused by the formation of protein complexes, which means that it may be caused by the existence of some protein complexes that are composed of a lot of proteins and overlap with other complexes. For example, the MIPS dataset consists of many complexes consisting of more than 10 proteins (*p*_*n*_>10), on the other hand, Aloy *et al*.’s dataset contains 98% complexes consisting of a lower number of proteins (*p*_*n*_≤10). Certainly, it is observed that the computation with MIPS dataset requires a lot of CPU time despite including a small number of complexes among three datasets, but it requires much less CPU time for the prediction with Aloy *et al*.’s dataset. Therefore, these results suggest that the CPU utilization depends not only on the number of complexes or proteins but also on the total number of proteins involved in one protein complex.

#### Results using known PPI datasets

**Fig 5:**
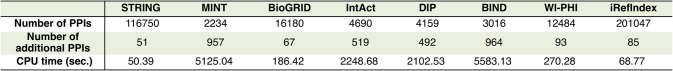
Summary of eight databases and results on MinPPI by GreedyMinPPI.

We examined GreedyMinPPI for MinPPI from known PPIs obtained from eight protein interaction databases such as STRING [15], MINT [16], BioGRID [17, 34], DIP [18], BIND [19, 35], WI-PHI [20], IntAct [21] and iRefIndex [23, 36], and using protein complexes in CYC2008 dataset. Although the same PPIs should be contained in different databases based on the different experimental techniques, known PPIs are limited to the extracted PPIs composed of 1627 proteins formed of CYC2008 protein complexes stored in these databases. The performance of GreedyMinPPI was evaluated by measuring the total number of additional interactions and the CPU time used to solve MinPPI, as shown in Fig 5.

It is shown that the minimum number of additional PPIs ranges from at least 51 (STRING) to 964 (BIND). In particular, in the case of BioGRID database, although it contains 16180 interactions, it is observed that 67 interactions are missing to support all interactions in the CYC2008. The results also mean that even the four largest PPI databases, STRING (51) in Table A4(a) in S1 File, BioGRID (67) in Table A7(a) in S1 File, WI-PHI (93) in Table A10(a) in S1 File and iRefIndex (85) in Table A13(a) in S1 File, do not have enough PPIs to form all CYC2008 protein complexes. For example, it is seen from Table A4 in S1 File that Dcs1p/Dcs2 heterodimer and Rad17p/Ddc1p/Mec3p complexes in CYC2008 are not fully covered by PPIs in STRING (Table A4(b) in S1 File) and GreedyMinPPI identifies 51 additional PPI pairs for supporting all CYC2008 complexes (Table A4(a) in S1 File). Table A5 in S1 File also presents the additional protein pairs and the complexes including each pair.

Furthermore, we analyzed the biological significance of additional protein pairs identified for four databases, STRING, BioGRID, WI-PHI and iRefIndex, using the PANTHER system (http://www.pantherdb.org/), which provides the classification of proteins and their genes according to family, subfamily, biological process, cellular component and molecular function. To evaluate the frequency distribution of the assigned categories, we counted the frequency in each category (see Tables A6, A9, A12 and A15 in S1 File). As for the molecular function categories, the top five across four databases are protein binding, oxidoreductase activity, RNA binding, pyrophosphatase activity and structural constituent of ribosome. However, from the PANTHER analysis, we could not observe a clear trend on the characteristic biological function for the additional proteins, which suggests that various types of complexes are not fully covered by the PPIs currently in the four databases.

In order to examine the plausibility of the predicted interactions, we performed in silico experiments. Among various excellent tools developed for prediction of protein interactions [37, 38], we employed PSOPIA [39] because it needs only sequence information, is easy to use, and is reported to have good prediction performance [39]. PSOPIA predicts the interaction between two protein sequences based on information from known homologous PPIs using Averaged One-Dependence Estimators. The interaction of two protein pairs is estimated by calculating the three features, sequence similarities (F_Seq_), statistical propensities (F_Dom_) and a sum of edge weights between homologous proteins (F_Net_). We performed the prediction for 67 protein pairs on BioGRID. Table A16 in S1 File summarizes the estimated scores for the additional protein pairs. Although the confidence is not high because of the high false positive rates in prediction of PPIs in PSOPIA and many other tools, it is found that many identified additional protein pairs possibly interact with each other (0.1 ≤ *S*_*all*_ < 0.5) and 8 among them were considered as highly probable (*S*_*all*_ ≥ 0.5). Furthermore, even if there is some data configuration change with real complex and PPI datasets, almost all approximate solutions are the same and the additional proteins are detected in the same order as mentioned in the prediction with synthetic data (see Table A2(b) in S1 File).

In addition, GreedyMinPPI only requires a small amount of CPU time regardless of the number of additional interactions and it might be effective for an application with large-scale datasets. To summarize, the current PPI data identified by eight databases is incomplete and does not adequately describe all PPIs involved in CYC2008; on the positive side, GreedyMinPPI enables us to detect the minimum number of additional interactions required to support all regulatory interactions among proteins which constitute the corresponding protein complex.

### Comparison of prediction performance

#### Performance comparison with weighted PPI datasets

To investigate the accuracy of GreedyMinPPI, we compared our results with those from existing PPI prediction methods using four different weighted PPI datasets predicted from Struct2Net [26], ENTS [27], PIP [28] and iWRAP [29]. Struct2Net is a web server for predicting PPIs based on the structural features using protein sequence data as input data. ENTS is a random forest based PPI prediction method only from the primary sequence data. PIP is developed based on a naïve Bayes classifier to stochastically predict whether each protein pair is present in the same complex regardless of their direct interaction. iWRAP is a threading-based prediction method for detecting de novo cancer related interactions. Since all interactions predicted from Struct2Net, ENTS, PIP and iWRAP were assigned the confidence score, we assessed by measuring the ROC curve and AUC (Area Under the Curve) score. In order to plot the ROC curve, we regarded the PPIs obtained from STRING, MINT, WI-PHI and IntAct databases as the gold standard (refer to S1 File) [40]. It must be noted that, in comparative experiments with those databases, we limited PPIs to those of extracted PPIs composed of 1627 proteins stored in those databases.

As for the confidence score assignment, for each interaction by GreedyMinPPI, the scores between [1-1344] were calculated to reflect the reliability where the firstly added interaction has the highest confidence which equals to 1344. Four existing methods also provided individual confidence scores, Struct2Net score ranging [0.25-0.98], ENTS score ranging [0.50-0.97], PIP score ranging [300.07-146673.28] and iWRAP score ranging [0.90-1.00].

**Fig 6:**
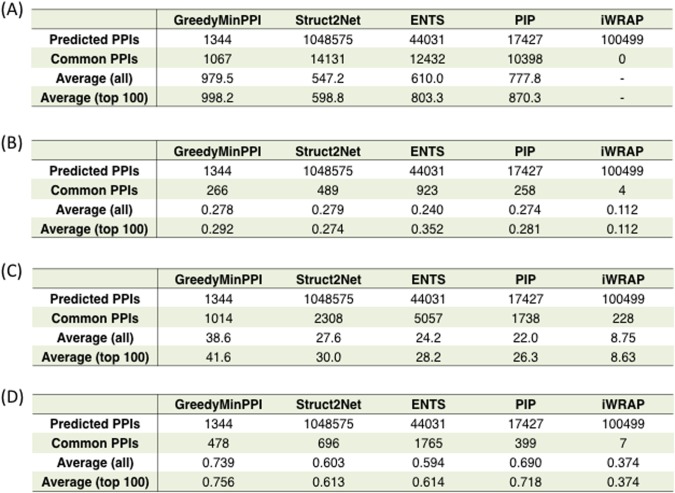
Comparison of the PPIs predicted by the five methods with STRING (A), MINT (B), WI-PHI (C) and IntAct (D) databases.

Fig 6 shows the number of predicted and common PPIs and the averages for all confidence scores and in the 100 highest scoring group for each database. The results show that the number of common PPIs that are shared between the PPIs predicted from GreedyMinPPI and those derived from STRING, WI-PHI are 1067, 1014, respectively, and the averages for all and the top 100 are the highest scoring among other methods. It should be noted that in case of IntAct, although the percentage of the number of common interactions is less than 50, the average for the top 100 by GreedyMinPPI is higher than those by other methods. It is because GreedyMinPPI has an advantage of being able to detect the minimum number of PPIs or additional PPIs by firstly adding edges among proteins that are most highly overlapping. In contrast, the averages of confidence scores of Struct2Net and ENTS are higher than those of GreedyMinPPI using MINT. Note also that since the iWRAP PPIs were predicted using the dataset of yeast cancer related genes and iWRAP detected an interaction between XPA (RAD14) and SMARCA5, whose overexpression leads to cell proliferation [29, 41], it provides only limited prediction and does not have many common interactions with four databases. In this way, although GreedyMinPPI does not guarantee the optimality of its solution, it enables us to provide high confidence protein interactions.

#### Comparison of distribution of PPI confidence score

**Fig 7:**
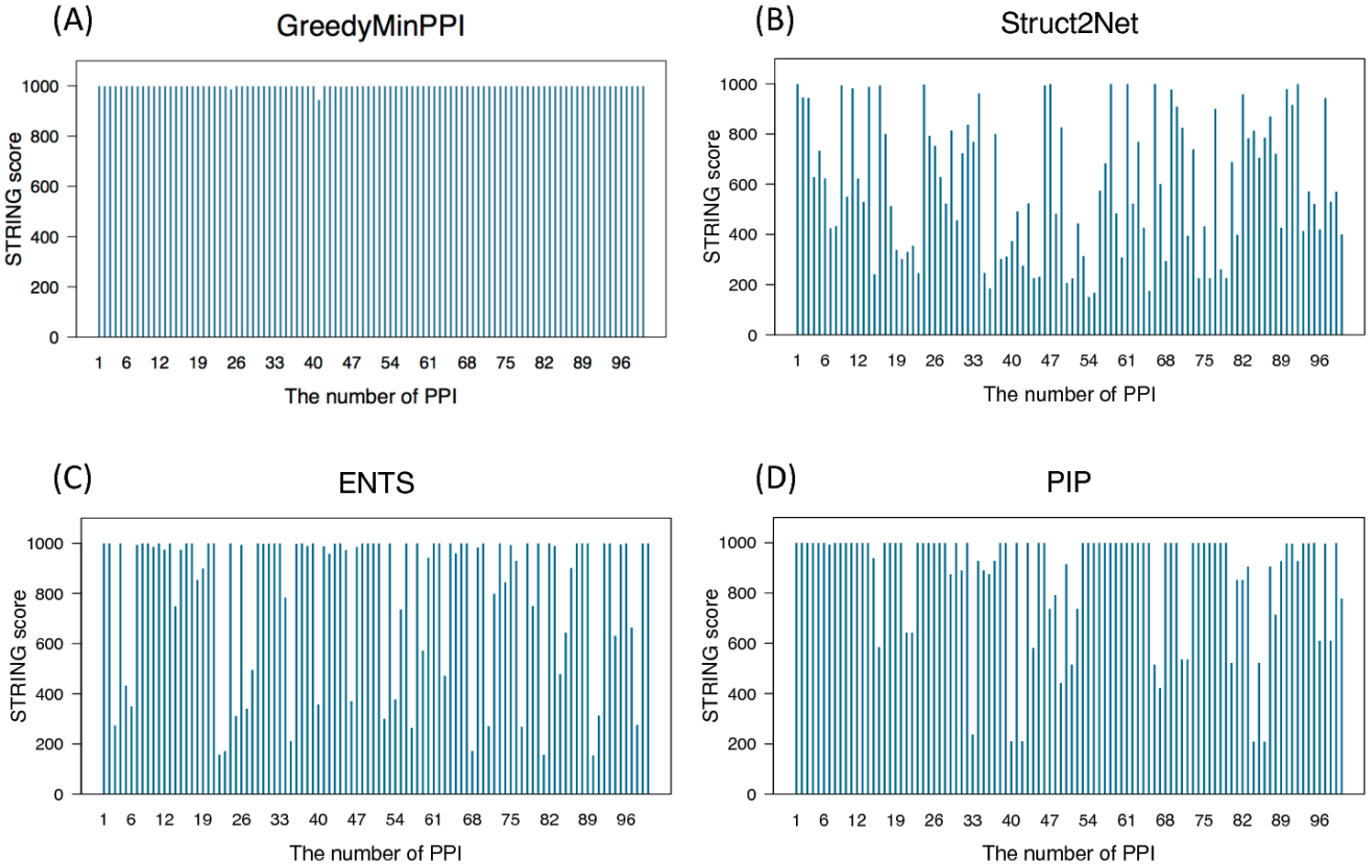
Distribution of PPI confidence score using STRING. The distribution of the confidence scores of PPIs predicted by GreedyMinPPI (A), Struct2Net (B), ENTS (C) and PIP (D).

Since the four databases provide confidence scores, each of which was computed by evidences from their own sources and all five scored PPIs were sorted in descending order in advance [42–44], we examined the distributions of database confidence score of the top 100 interactions that are calculated by five existing methods with STRING score ranging [150-999], MINT score ranging [0.091-0.984], WI-PHI score ranging [6.624-146.551] and IntAct score ranging [0.216-0.963] as shown in Fig 7 and A1–A4 Figs in S1 File.

In particular, the distribution with STRING exhibits that GreedyMinPPI detected the PPIs with uniformly high confidence score which suggests that the prediction is performed with a high reliability as shown by the high average score (Fig 7). Since iWRAP and STRING have no common edges, the distribution of iWRAP cannot be plotted. In case of WI-PHI, GreedyMinPPI can detect the interactions with relatively high confidence score than other methods, on the other hand, ENTS could predict interactions with higher scores than GreedyMinPPI using MINT. In case of IntAct, the distribution of the confidence score on GreedyMinPPI looks similar to the distribution by PIP, and certainly there is only a slight difference in the range of averages of top 100 interactions by GreedyMinPPI and PIP. Similarly, the distribution by Struct2Net and ENTS can appear to be almost the same (A1–A4 Figs in S1 File).

Therefore, the results suggest that the top 100 interactions predicted by GreedyMinPPI have higher confidence scores than those by existing methods but not providing the uniformly high scores except in the case of STRING. However, since it enables us to detect the interactions among proteins with most highly overlapping, most of these interactions would be expected to be more reliable.

#### Performance comparison with unweighted PPI datasets

In this subsection, we validated the predictive performance of GreedyMinPPI with three unweighted PPI prediction methods, PIPE [30], SPPS [45] and InteroPORC [46]. PIPE predicts PPIs only using information derived from the primary sequence of *S. cerevisiae* which consists of 6304 protein sequences. SPPS server is developed by combining Support Vector Machines (SVM) with the posterior probability which is derived from a sigmoid function, to search the possible interacting partners with the query protein sequence. InteroPORC provides an automated prediction based on the interolog concept, which reconstructs the orthology relationships across multiple species.

The performances of the three unweighted datasets were assessed by measuring six criteria, recall, precision, specificity, accuracy (ACC), *F*-measure and matthew’s correlation coefficient (MCC) (see S1 File). Recall is the proportion of actual positives which are correctly predicted; precision measures the proportion of positive predictions which are actual positives and there is a trade-off between recall and precision; specificity measures the proportion of actual negatives that are correctly predicted; accuracy is the proportion of the total number of correct predictions; *F*-measure is the harmonic mean of recall and precision; matthew’s correlation coefficient is the correlation coefficient between the actual and predicted binary classes, that takes from −1 to 1.

**Fig 8:**
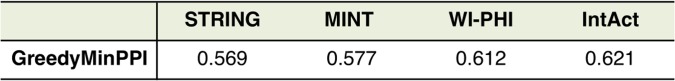
AUC scores on GreedyMinPPI with the four databases.

Needless to say, since the objective of MinPPI0 or MinPPI is to detect the minimum number of interactions or additional interactions so that all proteins belonging to each complex are connected, the number of predicted PPIs from GreedyMinPPI should mostly be lower than those from existing methods. This leads to the lower AUC performance of GreedyMinPPI compared to those of existing methods, as shown in Fig 8 and refer to the “original” in Tables A18–A21 in S1 File. Therefore, we tested whether the performance of existing methods can be improved by combining the interactions predicted from GreedyMinPPI. Firstly, we examined the six criteria of GreedyMinPPI and three methods using the four databases, STRING, MINT, WI-PHI and IntAct as the gold standard. Secondly, we also examined them to compare the performance of six criteria on their own datasets from PIPE, SPPS and InteroPORC with those on the combined datasets, each of which was constructed by adding all interactions from GreedyMinPPI. All measurement results are summarized in Table A17 in S1 File. Certainly, the four criteria except for specificity and ACC of GreedyMinPPI are relatively low for each database. However, almost all criteria of combined datasets for three existing methods are slightly improved in case of using STRING, MINT and WI-PHI databases, although some of them on the combined datasets perform a little bit worse than those on their own dataset using IntAct. From this experiment, it is found that since GreedyMinPPI predicts additional interactions including unknown PPIs, it might be helpful for enhancement of existing PPI prediction methods.

#### AUC performance comparison with weighted PPI datasets

To validate the performance enhancement of GreedyMinPPI with the weighted PPIs predicted from Struct2Net, ENTS, PIP and iWRAP in terms of AUC, we examined MinPPI using the dataset, combining all interactions and the corresponding confidence scores from GreedyMinPPI with those from the four existing methods. The combined scores were computed by,

$$\begin{array}{c} S_{c}=SPE\times S_{g}+S_{e} \end{array}$$ (3)

where *SPE* reflects the specific weight of scores predicted from GreedyMinPPI when combining the scores. *S*_*c*_ is the combined score and *S*_*g*_ and *S*_*e*_ are the individual scores that are calculated from GreedyMinPPI and existing methods, respectively.

Comparison results with the four databases are summarized in Tables A18–A21 in S1 File. Since iWRAP does not have any common edges with STRING, the AUC score of iWRAP on the original dataset could not be computed. In case of any database, regardless of the value of *SPE*, the AUC scores on the combined datasets increased than those on their own datasets from GreedyMinPPI. Because although GreedyMinPPI is based only on the connected components from graph theory, MinPPI tends to output pairs of proteins that are contained in many protein complexes. In other words, in this paper, we assume that the proteins consisting of highly overlapping probably interact with each other and these interactions might be missing interactions. In particular, the AUC on iWRAP was greatly improved when combining all interactions because fewer shared interactions exist in case of each database. On the other hand, the AUC performance was not improved using the combined datasets when combining top 200 and 500 interactions from GreedyMinPPI. These results suggest that GreedyMinPPI is also suitable for enhancement of the predictive performance of existing methods when all outputted interactions are utilized.

## Discussion and conclusion

In this paper, we have introduced MinPPI, which is a problem of determining the minimum number of PPIs required to support known protein complexes. For solving this problem, we have developed a novel integer linear programming-based method (ILPMinPPI) and a greedy-type method (GreedyMinPPI) based on an existing greedy-type approximation algorithm. The comparison of these two methods using moderate size synthetic data suggests that GreedyMinPPI outputs optimal or near-optimal solutions for practical instances. Since ILPMinPPI cannot be applied to large-scale data, we have applied GreedyMinPPI to pairs of a protein complex dataset and eight PPI datasets. Our findings show that the minimum number of additional required PPIs ranges from 51 (STRING) to 964 (BIND). Significantly, this suggests that even the four best PPI databases, STRING (51), BioGRID (67), WI-PHI (93) and iRefIndex (85) do not have enough PPIs to form all CYC2008 protein complexes. We have also applied GreedyMinPPI to enhance the existing PPI prediction methods. The results suggest that it is also useful for that purpose.

Although ILPMinPPI and GreedyMinPPI output PPIs with scores, they output PPIs only based on the minimum requirement that each complex must constitute a connected subgraph in a PPI network. Therefore, these methods are not optimized for prediction of PPIs and thus should be used only as auxiliary methods to enhance existing PPI prediction methods. However it might be possible to modify the formalization of MinPPI as a kind of machine-learning problem to infer PPIs from protein complexes. Although such a variant would still not be enough to be used as a stand-alone prediction method, it would be useful to further enhance the prediction accuracy of existing PPI prediction methods. Another important future work is to improve ILPMinPPI so that it can be applied to real protein complex datasets, because it is unclear whether GreedyMinPPI outputs near-optimal solutions for large-scale protein complex datasets and the theoretically guaranteed *O*(log *m*) approximation ratio is not enough to estimate the minimum number.

## Supporting information

S1 File Fig A1:Distribution of PPI confidence score using STRING. Fig A2:Distribution of PPI confidence score using MINT. Fig A3:Distribution of PPI confidence score using WI-PHI. Fig A4:Distribution of PPI confidence score using IntAct. Table A1:Performance evaluation of ILPMinPPI and GreedyMinPPI using synthetic data. Table A2:Prediction results on GreedyMinPPI with different data configuration. Table A3:Results with three protein complex datasets. Table A4:The additional protein pairs for supporting CYC2008 protein complex with STRING. Table A5:The additional protein pairs and the included complexes on STRING. Table A6, A9, A12, A15:Frequency distribution of the assigned categories of additional proteins on STRING from PANTHER analysis. Table A7:The additional protein pairs for supporting CYC2008 protein complex with BioGRID. Table A8:The additional protein pairs and the included complexes on BioGRID. Table A10:The additional protein pairs for supporting CYC2008 protein complex with WI-PHI. Table A11:The additional protein pairs and the included complexes on WI-PHI. Table A13:The additional protein pairs for supporting CYC2008 protein complex with iRefIndex. Table A14:The additional protein pairs and the included complexes on iRefIndex. Table A16:67 additional protein pairs on BioGRID and S_all_ which is the estimated score by PSOPIA. Table A17:Comparison of prediction performance with the four databases using unweighted datasets. Table A18:Comparison of prediction performance using AUC with STRING. Table A19:Comparison of prediction performance using AUC with MINT. Table A20:Comparison of prediction performance using AUC with WI-PHI. Table A21:Comparison of prediction performance using AUC with IntAct.
(PDF)
