# Supplementary material for: Determining the minimum number of protein-protein interactions required to support known protein complexes
Source: PLoS One. 2018 Apr 26;13(4):e0195545. doi: 10.1371/journal.pone.0195545 (PMC5919440; doi:10.1371/journal.pone.0195545)
Supplement: S1 File — Fig A1:Distribution of PPI confidence score using STRING. Fig A2:Distribution of PPI confidence score using MINT. Fig A3:Distribution of PPI confidence score using WI-PHI. Fig A4:Distribution of PPI confidence score using IntAct. Table A1:Performance evaluation of ILPMinPPI and GreedyMinPPI using synthetic data. Table A2:Prediction results on GreedyMinPPI with different data configuration. Table A3:Results with three protein complex datasets. Table A4:The additional protein pairs for supporting CYC2008 protein complex with STRING. Table A5:The additional protein pairs and the included complexes on STRING. Table A6, A9, A12, A15:Frequency distribution of the assigned categories of additional proteins on STRING from PANTHER analysis. Table A7:The additional protein pairs for supporting CYC2008 protein complex with BioGRID. Table A8:The additional protein pairs and the included complexes on BioGRID. Table A10:The additional protein pairs for supporting CYC2008 protein complex with WI-PHI. Table A11:The additional protein pairs and the included complexes on WI-PHI. Table A13:The additional protein pairs for supporting CYC2008 protein complex with iRefIndex. Table A14:The additional protein pairs and the included complexes on iRefIndex. Table A16:67 additional protein pairs on BioGRID and Sall which is the estimated score by PSOPIA. Table A17:Comparison of prediction performance with the four databases using unweighted datasets. Table A18:Comparison of prediction performance using AUC with STRING. Table A19:Comparison of prediction performance using AUC with MINT. Table A20:Comparison of prediction performance using AUC with WI-PHI. Table A21:Comparison of prediction performance using AUC with IntAct. (PDF) [file pone.0195545.s001.pdf]

Determining the Minimum Number of Protein-Protein Interactions Required to Support Known Protein Complexes

Natsu Nakajima[1], Morihiro Hayashida[2], Jesper Jansson[3], Osamu Maruyama[4] and Tatsuya Akutsu[5]

[1] Institute of Molecular and Cellular Biosciences, The University of Tokyo, 1-1-1, Yayoi, Bunkyo-ku, Tokyo 113-0032, Japan
[2] Department of Electrical Engineering and Computer Science, National Institute of Technology, Matsue College, 14-4, Nishiikumacho, Matsue, Shimane 690-8518, Japan
[3] Department of Computing, The Hong Kong Polytechnic University, Hung Hom, Kowloon, Hong Kong
[4] Institute of Mathematics for Industry, Kyushu University, 744 Motooka, Nishi-ku, Fukuoka 819-0395, Japan
[5] Bioinformatics Center, Institute for Chemical Research, Kyoto University, Gokasho, Uji, Kyoto 611-0011, Japan

April 5, 2018

Contents

1 Description of Weighted PPI Datasets 2

2 Computer Environment 2

3 Supplementary Figures 3

4 Supplementary Tables 7

1 Description of Weighted PPI Datasets

We examined MinPPI for eight databases of known protein interactions. Only four of them, STRING, MINT, IntAct and WI-PHI provide the reliability confidence score of known PPIs [41]. Since known PPIs are composed of many proteins, we prepared four datasets consisting of the interactions that are restricted to the 1627 proteins included in CYC2008 complexes.

- STRING database (Version 9.1) is a collection of known and predicted interactions, including both physical and functional associations covering about 5.0 million proteins in $\leq$ 1100 organisms and $\leq$ 200 million interactions. To extract the interactions of yeast, we take the downloadable STRING file (`http://string-db.org/download/protein.links.v10/4932.protein.links.v10.txt.gz`).

  Since STRING provides the combined score ranging [150-999], which reflects the reliability of each association, we regard them as the confidence score.

- MINT is one of the first protein interaction database and focuses on experimentally verified PPIs with the Yeast Two Hybrid (Y2H) [1], and Tandem Affinity Purification (TAP) [2] abstracted from literature-curated. The PPI data can be available from the website (`ftp://160.80.34.4/pub/release/txt/current/2012-10-29-mint-Sacchromyces.txt`).

  Since this data consists of several information about the protein interaction, in which particularly all interactions are assigned the reliability score ranging [0.091-0.984] according to the protein structure, sequence and function, we extract the protein name and the corresponding reliability score.

- IntAct provides the molecular interaction database which collects experimentally detected PPIs abstracted from the literature or the direct deposition of the data. The yeast protein interaction data can be downloaded from the website (`ftp://ebi.ac.uk/pub/database/intact/current/psimitab/intact-micluster.txt`) as a tab delimited files.

  Since this data contains the interaction information of various species including human, mouse, yeast, worm, etc., we extract a set of 4690 interacting protein pairs of yeast and the uniscore that refers to the reliability ranging [0.216-0.936], converting the UniProtKB/Swiss-Prot protein name into ORF name.

- WI-PHI provides the weighted physical interactions in the yeast proteome, in which the edge weight (reliability of the interaction) is computed based on the product of the socio-affinity index [3]. The data can be downloaded from the website (`http//www.wiley-vch.de/contents/jc-2120/2007/pro200600448_s.html/pro200600448_3_s.xls`).

  Since it contains a total of 50000 interacting protein pairs that are sorted according to the reliability scores ranging [6.624-146.551] in descending order, we generate and adopt the dataset consisting of 12484 protein interactions and the corresponding scores.

2 Computer Environment

The results shown in Fig 2, A1, A2, A3 and A4 Figs, Tables 1, 2, 3, 5, 6, A1,(syndata1, syndata2), A3, A17, A18, A19, A20 and A21 were performed using a PC with CPU 3.40 GHz and 7.7 GB RAM under Linux. The results shown in Tables 4, A1(syndata3, syndata4, syndata5), A2, A4, A5, A6, A7, A8, A9, A10, A11, A12, A13, A14, A15 and A16 were performed using a PC with CPU 3.30GHz$\times$8 and 62.9 GiB memory under Linux.

3 Supplementary Figures

we examined the distributions of database confidence score of the top 100 interactions that are calculated by five existing methods (GreedyMinPPI, Struct2Net, ENTS, PIP and iWRAP) with STRING score ranging [150-999], MINT score ranging [0.091-0.984], WI-PHI score ranging [6.624-146.551] and IntAct score ranging [0.216-0.963].

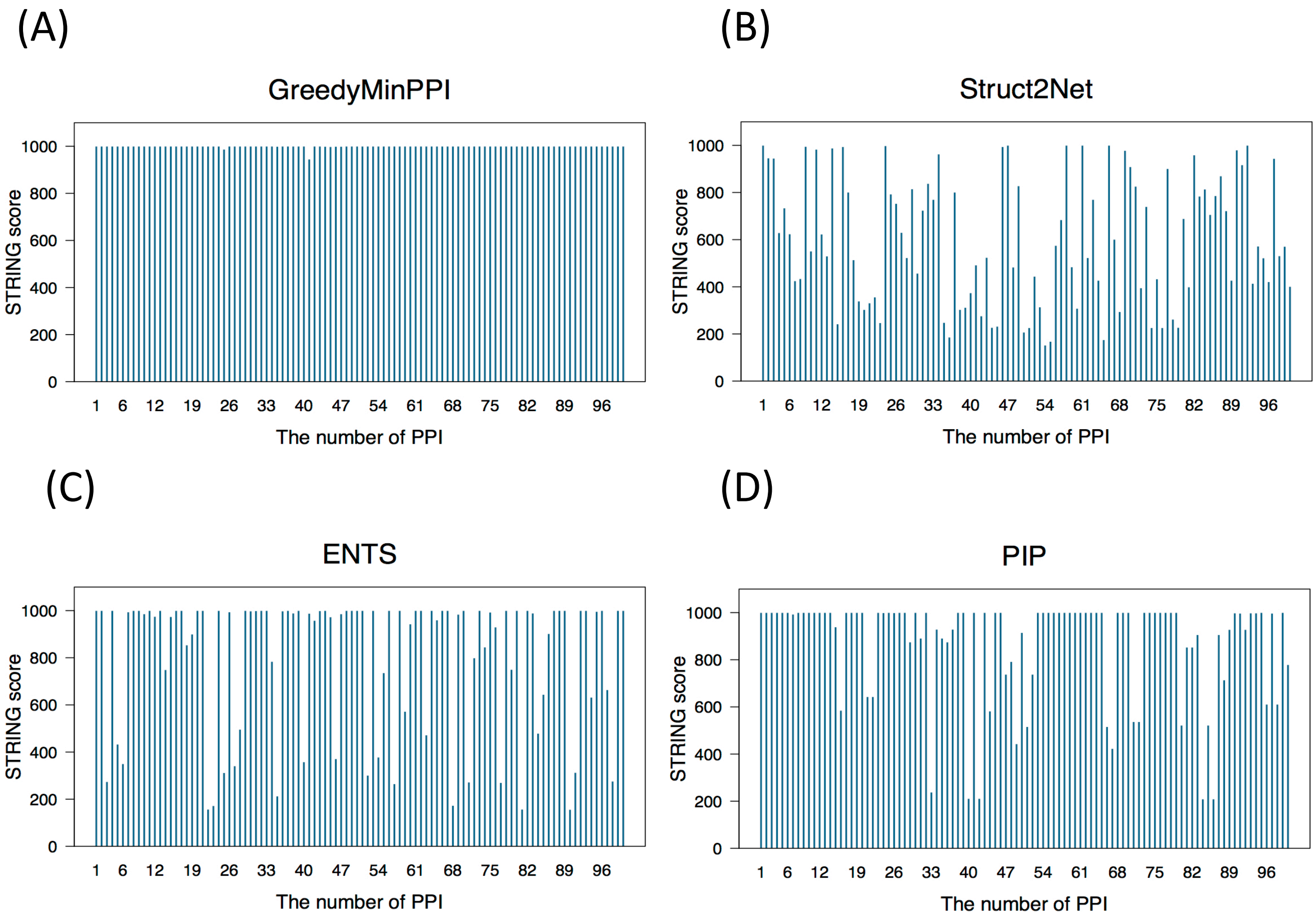

Figure A1: Distribution of PPI confidence score using STRING.
The distribution of the confidence scores of PPIs predicted by GreedyMinPPI (A), Struct2Net (B), ENTS (C) and PIP (D).

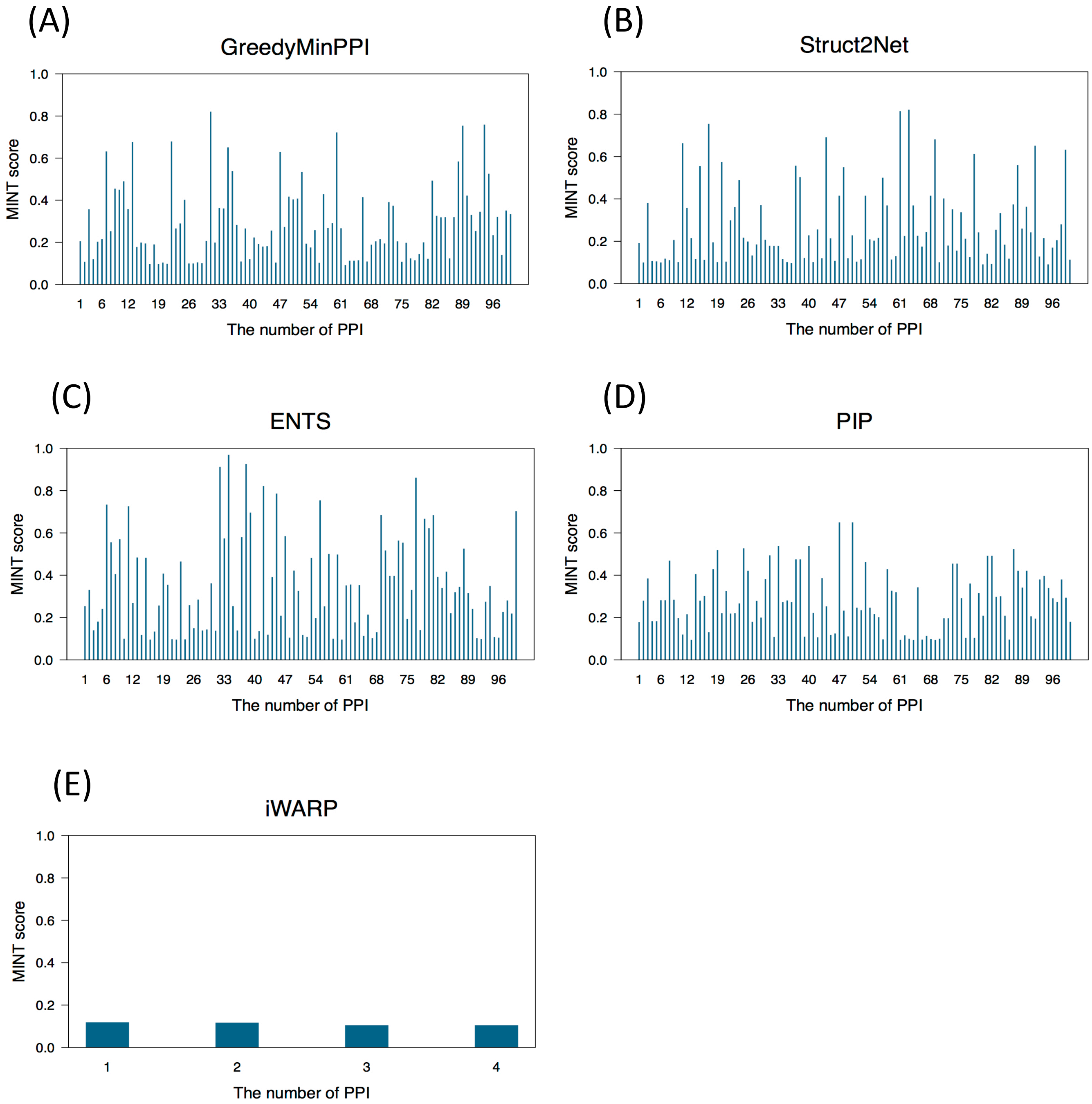

Figure A2: Distribution of PPI confidence score using MINT.
The distribution of the confidence scores of PPIs predicted by GreedyMinPPI (A), Struct2Net (B), ENTS (C), PIP (D) and iWARP (E).

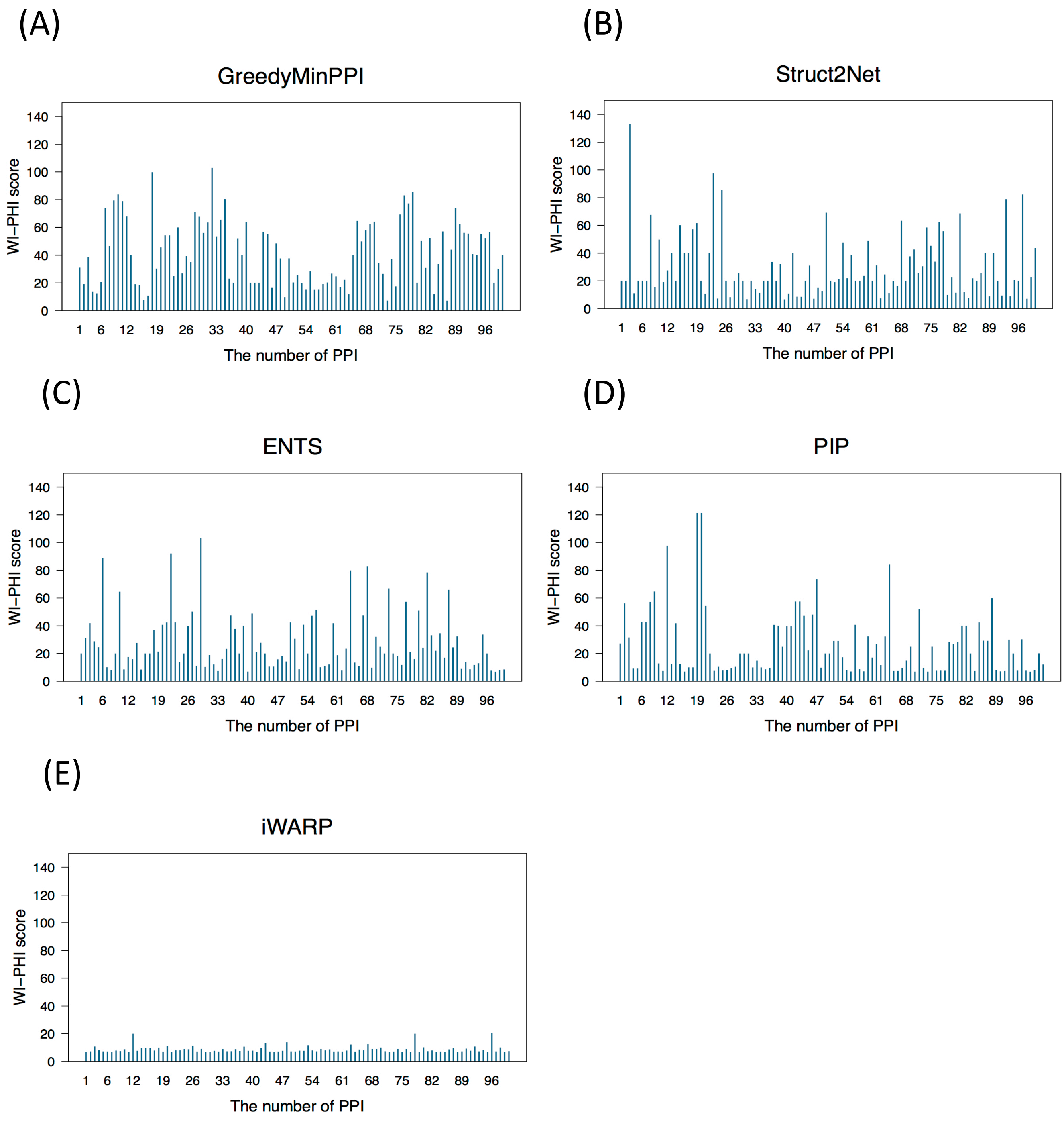

Figure A3: Distribution of PPI confidence score using WI-PHI.
The distribution of the confidence scores of PPIs predicted by GreedyMinPPI (A), Struct2Net (B), ENTS (C), PIP (D) and iWARP (E).

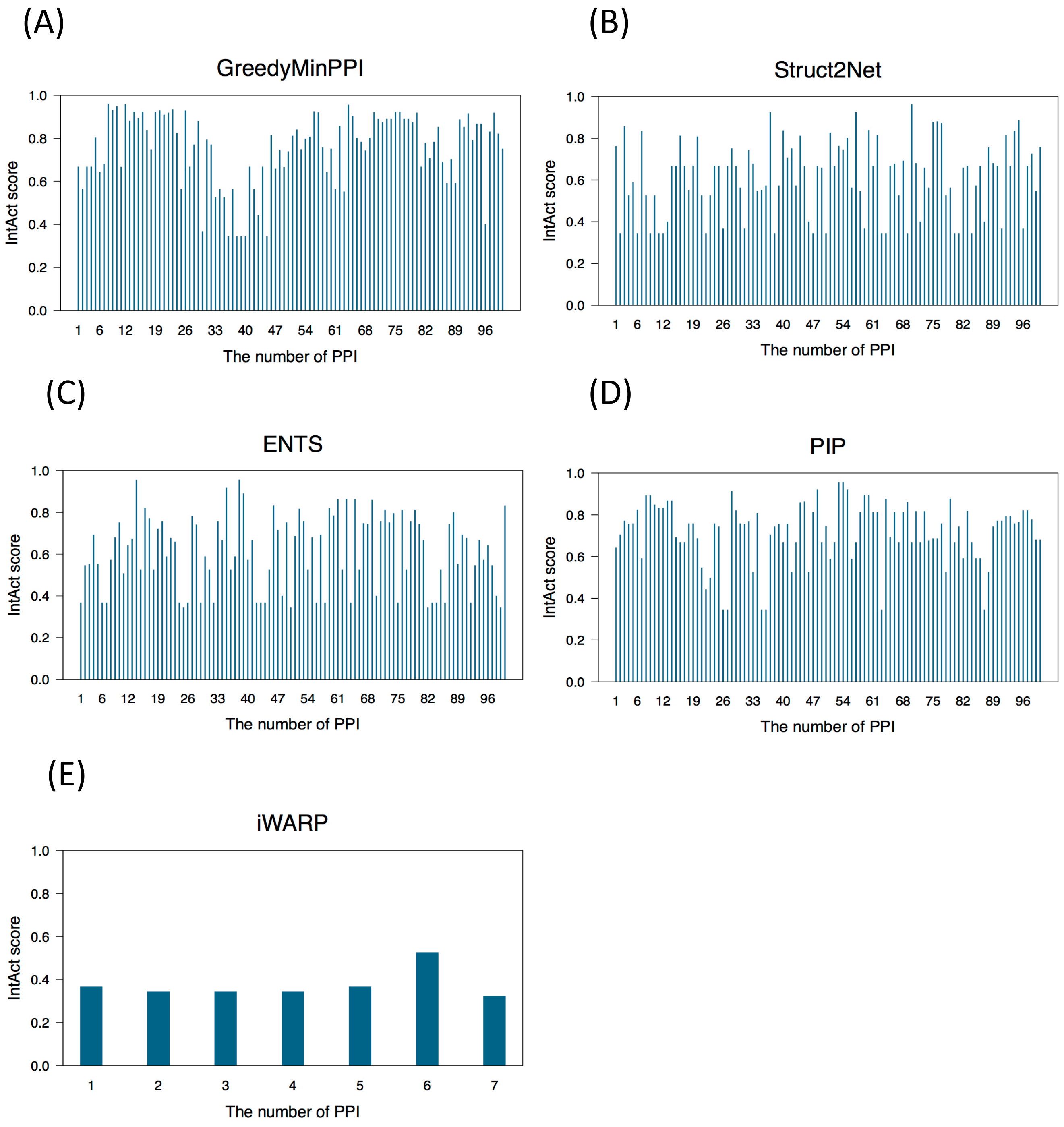

Figure A4: Distribution of PPI confidence score using IntAct.
The distribution of the confidence scores of PPIs predicted by GreedyMinPPI (A), Struct2Net (B), ENTS (C), PIP (D) and iWARP (E).

4 Supplementary Tables

We examined the performance evaluation of ILPMinPPI and GreedyMinPPI using synthetic data. We also examined GreedyMinPPI for MinPPI from known PPIs obtained from eight protein interaction databases using CYC2008 dataset. As for the four best databases, the additional protein pairs for supporting CYC2008 complex and the protein complexes that are not fully covered with each databse were summarized.

Table A1: Performance evaluation of ILPMinPPI and GreedyMinPPI using synthetic data.

(a) Summary of two synthetic datasets. syndata 1 is composed of 10 different datasets randomly generated, each of which contains a set of complexes where the maximum number of total proteins and complexes are 10 and 20, and the maximum number of proteins within a complex is 5.

	Maximum number of total proteins	Maximum number of total complexes	Maximum number of proteins within a complex
syndata 1	10	20	5
syndata 2	100	100	4
syndata 3	1600	400	5
syndata 4	1600	400	6
syndata 5	1600	400	15

(b) Results with syndata 1.

		data1	data2	data3	data4	data5	data6	data7	data8	data9	data10
ILPMinPPI	Number of outputted edges	2	13	16	15	14	4	16	13	5	19
	CPU time (sec.)	0.013	41.7	0.185	15.3	1.14	0.316	0.525	645.8	0.279	0.386
GreedyMinPPI	Number of outputted edges	2	13	16	16	14	4	25	13	5	19
	CPU time (10^{-3}sec.)	0.14	1.91	2.11	5.38	2.30	0.23	5.45	3.49	0.24	6.82
	Number of common PPIs	2	8	16	11	11	1	13	9	3	16

(c) Results with syndata 2.

		data1	data2	data3	data4	data5	data6	data7	data8	data9	data10
ILPMinPPI	Number of outputted edges	166	139	180	111	92	136	74	150	20	67
	CPU time (sec.)	9.36	9.44	10.7	7.53	5.51	9.05	5.09	12.3	1.45	4.74
GreedyMinPPI	Number of outputted edges	166	139	180	111	92	136	74	150	20	67
	CPU time (sec.)	1.14	0.651	1.45	0.341	0.206	0.606	0.141	0.755	0.00941	0.107
	Rate of common PPIs (%)	65.7	63.3	63.3	5.41	71.7	46.3	58.1	46.0	80.0	70.1

(d) Results with syndata 3.

		data1	data2	data3	data4	data5	data6	data7	data8	data9	data10
ILPMinPPI	Number of outputted edges	920	460	226	913	858	839	792	253	586	955
	CPU time (sec.)	40.5	14.1	5.96	17.0	35.0	37.0	35.7	12.5	15.0	43.0
GreedyMinPPI	Number of outputted edges	920	460	226	913	858	839	792	253	586	955
	CPU time (sec.)	210.9	32.19	7.969	201.5	169.0	163.3	132.6	11.2	61.89	231.5
	Rate of common PPIs (%)	47.0	58.5	53.5	63.6	51.0	54.1	52.7	49.0	53.4	55.4

(e) Results with syndata 4.

		data1	data2	data3	data4	data5	data6	data7	data8	data9	data10
ILPMinPPI	Number of outputted edges	307	503	582	908	774	736	894	849	1082	1172
	CPU time (sec.)	33.57	72.00	96.57	128.8	113.8	122.9	121.3	130.5	156.0	250.0
GreedyMinPPI	Number of outputted edges	307	503	582	908	774	736	894	849	1082	1172
	CPU time (sec.)	17.81	51.46	80.09	293.0	160.6	162.1	283.3	241.5	482.2	614.2
	Rate of common PPIs (%)	40.7	43.9	57.0	39.9	42.9	49.9	41.6	39.3	44.5	46.0

(f) Results with syndata 5.

		data1	data2	data3	data4	data5	data6	data7	data8	data9	data10
GreedyMinPPI	Number of outputted edges	2421	2294	2710	2509	2383	2693	1608	2511	1856	1495
	CPU time (sec.)	16603.9	14440.4	29240.6	19205.7	17842.4	25484.9	2162.27	20168.7	8347.36	5090.60

Table A2 : Prediction results on GreedyMinPPI with different data configuration.

(a) Results with synthetic data.

	data1	data2	data3	data4	data5	data6	data7	data8	data9	data10
Number of complexes	331	83	228	312	309	356	259	255	400	392
Number of proteins	1600	1600	1600	1597	1599	1596	1597	1600	1600	1600
Number of outputted edges	831	200	567	740	748	918	643	638	990	963
CPU time (sec.)	148.4	7.148	55.92	108.7	116.1	212.3	77.34	76.48	245.8	238.6

(b) Results with CYC2008 complex data. The different configuration data was generated by converting through the shuffle for the CYC2008 and known PPIs from eight databases each 10 times. STRING outputted another approximate solution in 9 times out of 100.

	STRING	MINT	BioGRID	IntAct	DIP	BIND	WI-PHI	iRefIndex
Number of outputted edges	51 (139)	957	67	519	492	964	93	85
CPU time (sec.)	53.913 (80.898)	5101.6	182.84	2197.2	2056.4	5402.0	259.90	69.417

Table A3 : Results with three protein complex datasets.

(a) Summary of three protein complex datasets and the number of outputted edges by GreedyMinPPI.

	Number of complexes	Number of proteins	Number of outputted edges	CPU time (sec.)
CYC2008	408	1627	1344	9212.3
MIPS	203	1189	1154	62103.0
Aloy *et al.*	468	1008	772	628.4

(b) Summary of the number of complexes composed of p_n proteins. p_n is the number of proteins involved in one protein complex.

	$p_n \leq 10$	$10 < p_n \leq 20$	$20 < p_n \leq 30$	$30 < p_n \leq 40$	$40 < p_n \leq 50$	$50 < p_n \leq 60$	$60 < p_n \leq 70$	$70 < p_n \leq 80$	$80 < p_n \leq 90$	$90 < p_n \leq 100$
CYC2008	376	24	4	1	1	1	0	0	1	0
MIPS	137	35	12	8	2	3	0	4	1	1
Aloy *et al.*	462	6	0	0	0	0	0	0	0	0

Table A4 : The additional protein pairs for supporting CYC2008 protein complex and the protein complexes that are not fully covered with STRING.

(a) The additional protein pairs for supporting CYC2008 complexes when examining GreedyMinPPI from known PPI on STRING.

Name of gene encoding the protein on SGD[1]	
DCS1	DCS2
DDC1	MEC3
DDC1	RAD17
ELC1	ELA1
TRM11	TRM112
MLH1	MLH3
MLH1	PMS1
MLH1	MLH2
ERF2	SHR5
PXA1	PXA2
PSR1	WHI2
BUR2	SGV1
YKU70	YKU80
GAL4	GAL80
DCP1	DCP2
PCL8	PHO85
PHO85	PHO80
PHO85	PCL1
MDM20	NAT3
HRD1	HRD3
FKS1	RHO1
MNN9	VAN1
SNO1	SNZ1
GYL1	GYP5
IDH1	IDH2
FAS1	FAS2
RTT109	VPS75
RPD3	SIN3
RPD3	UME1
RPD3	EAF3
RPD3	RCO1
RSE1	HSH155
RSE1	HSH49
RSE1	CUS1
RSE1	YSF3
RSE1	RDS3
GCR1	GCR2
DSS1	SUV3
NOC2	NOC3
SUI1	TIF11
RAD1	RAD10
RAD1	RAD14
UBA3	ULA1
PBI2	TRX1
MEX67	MTR2
TOA1	TOA2
FIG4	VAC14
RMI1	SGS1
RMI1	TOP3
CMP2	CNA1
CMP2	CNB1

(b) The list of the protein complexes which are not fully covered with STRING.

Name of the protein complex
Dcs1p/Dcs2 heterodimer
Rad17p/Ddc1p/Mec3p complex
nucleotide-excision repair factor 4 complex
Ela1p/Elc1p complex
Cul3p-RING ubiquitin ligase complex
adoMet-dependent tRNA methyltr ansferase (Mtase) Complex
Mlh1p/Mlh3p complex
Mlh1p/Pms1p complex
Mlh1p/Mlh2p complex
palmitoyltransferase complex
Pxa1p/Pxa2p complex
Psr1p/Whi2p complex
Sgv1p/Bur2p complex
Ku complex
GAL4p/GAL80p complex
GAL3p/GAL80p complex
Decapping Enzyme Complex
Pho85p/Pcl8p complex
Pho85p/Pho80p complex
Pho85p/Pcl10p complex
Pho85p/Pcl1p complex
Pho85p/Pcl2p complex
NatB complex
ubiquitin ligase ERAD-L complex
ubiquitin ligase ERAD-M complex
luminal surveillance complex
1,3-beta-glucan synthase complex (Fks1p/Rho1p)
alpha-1,6-mannosyltransferase complex(Van1p/Mnn9p)
alpha-1,6-mannosyltransferase complex(Anp1p/Mnn9p)
Sno1p/Snz1p complex
Gyl1p/Gyp5p complex
Isocitrate dehydrogenase
fatty acid synthase complex
Rtt109p/Vps75p complex
Rpd3L complex
Rpd3S complex
Rpd3L complex
Rpd3S complex
NuA4 histone acetyltransferase complex
U2 snRNP complex
SF3b complex
GCR complex
degradosome
Noc2p/Noc3p complex
Noc1p/Noc2p complex
eIF1/eIF1A/40S complex
multi-eIF complex
nucleotide-excision repair factor 1 complex
Ula1p/Uba3p complex
LMA1 complex
Mtr2p/Mex67p complex
transcription factor TFIIA complex
Fig4p/Vac14p complex
RecQ helicase-Topo III complex
calcineurin complex

[1] Saccharomyces GENOME DATABASE

Table A5 : The additional protein pairs and the included complexes on STRING. These proteins correspond to the protein pairs described in Table A4(a). For example, The name of the gene encording the protein numbering 127 is ELC1 which is a subunit of the following complexes, nucleotide-excision repair factor 4 complex, Ela1p/Elc1p complex and Cul3p-RING ubiquitin ligase complex.

99	100	99: Dcs1p/Dcs2 heterodimer<br>100: Dcs1p/Dcs2 heterodimer
113	114	113: Rad17p/Ddc1p/Mec3p complex<br>114: Rad17p/Ddc1p/Mec3p complex
113	115	113: Rad17p/Ddc1p/Mec3p complex<br>115: Rad17p/Ddc1p/Mec3p complex
127	193	127: nucleotide-excision repair factor 4 complex<br>127: Ela1p/Elc1p complex<br>127: Cul3p-RING ubiquitin ligase complex<br>193: Ela1p/Elc1p complex<br>193: Cul3p-RING ubiquitin ligase complex
133	134	133: adoMet-dependent tRNA methyltransferase (Mtase) Complex<br>134: adoMet-dependent tRNA methyltransferase (Mtase) Complex
154	155	154: Mlh1p/Mlh3p complex<br>154: Mlh1p/Pms1p complex<br>154: Mlh1p/Mlh2p complex<br>155: Mlh1p/Mlh3p complex
154	1089	154: Mlh1p/Mlh3p complex<br>154: Mlh1p/Pms1p complex<br>154: Mlh1p/Mlh2p complex<br>1089: Mlh1p/Pms1p complex
154	1338	154: Mlh1p/Mlh3p complex<br>154: Mlh1p/Pms1p complex<br>154: Mlh1p/Mlh2p complex<br>1338: Mlh1p/Mlh2p complex
209	210	209: palmitoyltransferase complex
304	305	304: Pxa1p/Pxa2p complex<br>305: Pxa1p/Pxa2p complex
332	333	332: Psr1p/Whi2p complex<br>333: Psr1p/Whi2p complex
372	373	372: Sgv1p/Bur2p complex<br>373: Sgv1p/Bur2p complex
425	426	425: Ku complex<br>426: Ku complex
429	430	429: GAL4p/GAL80p complex<br>430: GAL4p/GAL80p complex<br>430: GAL3p/GAL80p complex
440	441	440: Decapping Enzyme Complex<br>441: Decapping Enzyme Complex
615	616	615: Pho85p/Pcl8p complex<br>616: Pho85p/Pcl8p complex<br>616: Pho85p/Pho80p complex<br>616: Pho85p/Pcl10p complex<br>616: Pho85p/Pcl1p complex<br>616: Pho85p/Pcl2p complex
616	1187	616: Pho85p/Pcl8p complex<br>616: Pho85p/Pho80p complex<br>616: Pho85p/Pcl10p complex<br>616: Pho85p/Pcl1p complex<br>616: Pho85p/Pcl2p complex<br>1187: Pho85p/Pho80p complex
616	1492	616: Pho85p/Pcl8p complex<br>616: Pho85p/Pho80p complex<br>616: Pho85p/Pcl10p complex<br>616: Pho85p/Pcl1p complex<br>616: Pho85p/Pcl2p complex<br>1492: Pho85p/Pcl1p complex
661	662	661: NatB complex<br>662: NatB complex
673	674	673: ubiquitin ligase ERAD-L complex<br>673: ubiquitin ligase ERAD-M complex<br>674: ubiquitin ligase ERAD-L complex<br>674: luminal surveillance complex<br>674: ubiquitin ligase ERAD-M complex
721	722	721: 1,3-beta-glucan synthase complex (Fks1p/Rho1p)<br>722: 1,3-beta-glucan synthase complex (Fks1p/Rho1p)
788	789	788: alpha-1,6-mannosyltransferase complex(Van1p/Mnn9p)<br>788: alpha-1,6-mannosyltransferase complex(Anp1p/Mnn9p)<br>789: alpha-1,6-mannosyltransferase complex(Van1p/Mnn9p)
806	807	806: Sno1p/Snz1p complex<br>807: Sno1p/Snz1p complex
809	810	809: Gyl1p/Gyp5p complex<br>810: Gyl1p/Gyp5p complex
827	828	827: Isocitrate dehydrogenase<br>828: Isocitrate dehydrogenase
829	830	829: fatty acid synthase complex<br>830: fatty acid synthase complex
838	839	838: Rtt109p/Vps75p complex<br>839: Rtt109p/Vps75p complex
848	853	848: Rpd3L complex<br>848: Rpd3S complex<br>853: Rpd3L complex<br>853: Rpd3S complex
848	854	848: Rpd3L complex<br>848: Rpd3S complex<br>854: Rpd3L complex<br>854: Rpd3S complex
848	1122	848: Rpd3L complex<br>848: Rpd3S complex<br>1122: NuA4 histone acetyltransferase complex<br>1122: Rpd3S complex
848	1208	848: Rpd3L complex<br>848: Rpd3S complex<br>1208: Rpd3S complex
857	858	857: U2 snRNP complex<br>857: SF3b complex<br>858: U2 snRNP complex<br>858: SF3b complex
857	859	857: U2 snRNP complex<br>857: SF3b complex<br>859: U2 snRNP complex<br>859: SF3b complex
857	860	857: U2 snRNP complex<br>857: SF3b complex<br>860: U2 snRNP complex
857	1118	857: U2 snRNP complex<br>857: SF3b complex<br>1118: SF3b complex
857	1119	857: U2 snRNP complex<br>857: SF3b complex<br>1119: SF3b complex
892	893	892: GCR complex<br>893: GCR complex
933	934	933: degradosome<br>934: degradosome
1037	1038	1037: Noc2p/Noc3p complex<br>1037: Noc1p/Noc2p complex<br>1038: Noc2p/Noc3p complex
1099	1100	1099: eIF1/eIF1A/40S complex<br>1099: multi-eIF complex<br>1100: eIF1/eIF1A/40S complex
1156	1157	1156: nucleotide-excision repair factor 1 complex<br>1157: nucleotide-excision repair factor 1 complex
1156	1158	1156: nucleotide-excision repair factor 1 complex<br>1158: nucleotide-excision repair factor 1 complex
1269	1270	1269: Ula1p/Uba3p complex<br>1270: Ula1p/Uba3p complex
1328	1329	1328: LMA1 complex<br>1329: LMA1 complex
1365	1366	1365:Mtr2p/Mex67p complex<br>1366:Mtr2p/Mex67p complex
1369	1370	1369: transcription factor TFIIA complex<br>1370: transcription factor TFIIA complex
1460	1461	1460: Fig4p/Vac14p complex<br>1461: Fig4p/Vac14p complex
1489	1490	1489: RecQ helicase-Topo III complex
1489	1491	1489: RecQ helicase-Topo III complex<br>1491: RecQ helicase-Topo III complex
1571	1572	1571: calcineurin complex
1571	1573	1571: calcineurin complex<br>1573: calcineurin complex

Table A6: Frequency distribution of the assigned categories of additional proteins on STRING from PANTHER analysis, (a) Biological process (b) Cellular component (c) Molecular function. FQ shows the count for each matched category.

(a) 70 categories of biological process.

Category of biological process	FQ
cellular process	42
nitrogen compound metabolic process	36
catabolic process	16
biosynthetic process	15
response to stress	14
DNA repair	13
phosphate-containing compound metabolic process	12
cellular component biogenesis	7
regulation of transcription from RNA polymerase II promoter	6
cell cycle	5
mRNA splicing, via spliceosome	5
RNA catabolic process	5
RNA splicing, via transesterification reactions	5
chromatin organization	5
organelle organization	5
cellular protein modification process	4
DNA recombination	4
protein localization	4
regulation of cell cycle	4
protein acetylation	4
regulation of phosphate metabolic process	3
carbohydrate metabolic process	3
proteolysis	3
regulation of biological process	3
reproduction	3
protein targeting	3
response to abiotic stimulus	3
protein lipidation	2
transcription elongation from RNA polymerase II promoter	2
vitamin biosynthetic process	2
nuclear transport	2
rRNA metabolic process	2
nucleobase-containing compound metabolic process	2
meiosis	2
cellular component organization	2
coenzyme metabolic process	2
lipid metabolic process	2
homeostatic process	2
generation of precursor metabolites and energy	2
tricarboxylic acid cycle	2
lipid transport	2
regulation of catalytic activity	1
cellular component morphogenesis	1
transcription initiation from RNA polymerase II promoter	1
fatty acid biosynthetic process	1
phospholipid metabolic process	1
cytokinesis	1
cellular amino acid metabolic process	1
DNA replication	1
cytoskeleton organization	1
locomotion	1
localization	1
regulation of gene expression, epigenetic	1
intracellular signal transduction	1
regulation of sequence-specific DNA binding transcription factor activity	1
glycolysis	1
protein glycosylation	1
regulation of carbohydrate metabolic process	1
transcription from RNA polymerase II promoter	1
protein methylation	1
nucleobase-containing compound transport	1
vesicle-mediated transport	1
tRNA metabolic process	1
RNA localization	1
mitochondrion organization	1
regulation of nucleobase-containing compound metabolic process	1
chromosome segregation	1
intracellular protein transport	1
response to stimulus	1
cellular component movement	1

(b) 22 categories of cellular component.

Category of cellular component	FQ
protein complex	33
intracellular	31
nucleus	24
cytoplasm	15
nucleoplasm	8
ribonucleoprotein complex	8
nuclear chromosome	8
cytosol	3
chromosome	2
vacuole	2
integral to membrane	2
Golgi apparatus	2
organelle	2
integral to membrane	2
nuclear envelope	1
endoplasmic reticulum	1
nucleolus	1
mitochondrion	1
endosome	1
membrane	1
plasma membrane	1
cell part	1

(c) 42 categories of molecular function.

Category of molucular function	FQ
pyrophosphatase activity	10
single-stranded DNA binding	6
oxidoreductase activity	4
RNA binding	4
protein binding	4
protein kinase activity	3
damaged DNA binding	3
transmembrane transporter activity	2
deacetylase activity	2
nucleic acid binding	2
transcription cofactor activity	2
transferase activity, transferring acyl groups	2
mRNA binding	2
endodeoxyribonuclease activity	2
catalytic activity	2
DNA binding	2
acetyltransferase activity	2
double-stranded DNA binding	2
GTPase activity	2
hydrolase activity	2
nucleotide binding	1
phosphatase inhibitor activity	1
DNA helicase activity	1
phosphoprotein phosphatase activity	1
kinase activity	1
enzyme regulator activity	1
transporter activity	1
peptidase inhibitor activity	1
kinase regulator activity	1
enzyme activator activity	1
RNA polymerase II transcription factor binding transcription factor activity	1
RNA methyltransferase activity	1
ubiquitin-protein ligase activity	1
nuclease activity	1
signal transducer activity	1
translation initiation factor activity	1
chromatin binding	1
transferase activity, transferring glycosyl groups	1
RNA helicase activity	1
exoribonuclease activity	1
sequence-specific DNA binding RNA polymerase II transcription factor activit	1
lyase activity	1

Table A7 : The additional protein pairs for supporting CYC2008 protein complex and the protein complexes that are not fully covered with BioGRID.

(a) The additional protein pairs for supporting CYC2008 complexes when examining GreedyMinPPI from known PPI on BioGRID.

Name of gene encoding the protein	
SBH2	SSH1
SBH2	SSS1
MSS1	MTO1
INH1	STF2
INH1	ATP19
SMB1	PRP28
HOP1	ZIP2
IMG1	MRPL31
IMG1	MRPL33
IMG1	MRPL38
IMG1	MRPL49
DYN1	DYN3
LPD1	GCV1
LPD1	GCV2
LPD1	GCV3
ARG2	ARG5,6
COB	COR1
COB	QCR10
COB	QCR7
COB	QCR8
COB	QCR9
MET10	MET5
RSE1	YSF3
COX1	COX12
COX1	COX13
MGE1	PAM17
DSS1	SUV3
RPL10	RPL14A
RPL10	RPL15B
RPL10	RPL21B
RPL10	RPL22A
RPL10	RPL22B
RPL10	RPL34A
RPL10	RPL34B
RPL10	RPL37A
RPL10	RPL37B
RPL10	RPL39
RPL10	RPL40A
RPL10	RPL40B
RPL10	RPL41C
RPL10	RPL41B
RPL10	RPL42A
RPL10	RPS43A
SDH1	SDH3
PBI2	TRX1
ASC1	RPS10A
ASC1	RPS12
ASC1	RPS16A
ASC1	RPS19A
ASC1	RPS21A
ASC1	RPS21B
ASC1	RPS23B
ASC1	RPS26A
ASC1	RPS27A
ASC1	RPS27B
ASC1	RPS28A
ASC1	RPS29A
ASC1	RPS29B
ASC1	RPS30B
ASC1	RPS31
ERI1	GPI1
ERI1	GPI15
ERI1	GPI19
TOM20	TOM6
HSC82	SRO9
MRP1	PPE1
MRP1	YMR31

(b) The list of the protein complexes that are not fully covered with BioGRID.

Name of the protein complex
Ssh1p translocon complex
Sec61p translocon complex
Mto1p/Mss1p complex
mitochondrial ATPase inhibitor complex
F0/F1 ATP synthase (complex V)
commitment complex
U5 snRNP complex
U4/U6 x U5 tri-snRNP complex
U2 snRNP complex
U1 snRNP complex
Synaptonemal complex (SC)
mitochondrial ribosomal large subunit
cytoplasmic dynein complex
mitochondrial oxoglutarate dehydrogenase complex
glycine cleavage complex
mitochondrial pyruvate dehydrogenase complex
Arg2p/Arg5,6p complex
Cytochrome bc1 complex (Ubiquinol-cytochrome c reductase complex, complex III)
sulfite reductase complex (NADPH)
U2 snRNP complex
SF3b complex
Cytochrome c oxidase (complex IV)
Cox14p/Cox1p/Mss51p complex
PAM complex
degradosome
cytoplasmic ribosomal large subunit
Succinate dehydrogenase complex (complex II)
LMA1 complex
cytoplasmic ribosomal small subunit
glycosylphosphatidylinositol-N-acetylglucosaminyltransferase (GPI-GnT) complex
mitochondrial outer membrane translocase complex
HMC Complex
mitochondrial ribosomal small subunit

Table A8: The additional protein pairs and the included complexes on BioGRID. These proteins correspond to the protein pairs described in Table A7(a). For example, The name of the gene encording the protein numbering 218 is INH1 which is a subunit of the following complexes, mitochondrial ATPase inhibitor complex and F0/F1 ATP synthase (complex V).

45	46	45: Ssh1p translocon complex<br>46: Ssh1p translocon complex
45	47	45: Ssh1p translocon complex<br>47: Ssh1p translocon complex<br>47: Sec61p translocon complex
57	58	57: Mto1p/Mss1p complex<br>58: Mto1p/Mss1p complex
218	220	218: mitochondrial ATPase inhibitor complex<br>218: F0/F1 ATP synthase (complex V)<br>220: mitochondrial ATPase inhibitor complex
218	516	218: mitochondrial ATPase inhibitor complex<br>218: F0/F1 ATP synthase (complex V)<br>516: F0/F1 ATP synthase (complex V)
235	578	235: commitment complex<br>235: U5 snRNP complex<br>235: U4/U6 x U5 tri-snRNP complex<br>235: U2 snRNP complex<br>235: U1 snRNP complex<br>578: U5 snRNP complex
431	434	431: Synaptonemal complex (SC)<br>434: Synaptonemal complex (SC)
527	549	527: mitochondrial ribosomal large subunit<br>549: mitochondrial ribosomal large subunit
527	551	527: mitochondrial ribosomal large subunit<br>551: mitochondrial ribosomal large subunit
527	555	527: mitochondrial ribosomal large subunit<br>555: mitochondrial ribosomal large subunit
527	560	527: mitochondrial ribosomal large subunit<br>560: mitochondrial ribosomal large subunit
617	619	617: cytoplasmic dynein complex<br>619: cytoplasmic dynein complex
656	889	656: mitochondrial oxoglutarate dehydrogenase complex<br>656: glycine cleavage complex<br>656: mitochondrial pyruvate dehydrogenase complex<br>889: glycine cleavage complex
656	890	656: mitochondrial oxoglutarate dehydrogenase complex<br>656: glycine cleavage complex<br>656: mitochondrial pyruvate dehydrogenase complex<br>890: glycine cleavage complex
656	891	656: mitochondrial oxoglutarate dehydrogenase complex<br>656: glycine cleavage complex<br>656: mitochondrial pyruvate dehydrogenase complex<br>891: glycine cleavage complex
717	718	717: Arg2p/Arg5,6p complex<br>718: Arg2p/Arg5,6p complex
760	761	760: Cytochrome bc1 complex (Ubiquinol-cytochrome c reductase complex, complex III)<br>761: Cytochrome bc1 complex (Ubiquinol-cytochrome c reductase complex, complex III)
760	763	760: Cytochrome bc1 complex (Ubiquinol-cytochrome c reductase complex, complex III)<br>763: Cytochrome bc1 complex (Ubiquinol-cytochrome c reductase complex, complex III)
760	766	760: Cytochrome bc1 complex (Ubiquinol-cytochrome c reductase complex, complex III)<br>766: Cytochrome bc1 complex (Ubiquinol-cytochrome c reductase complex, complex III)
760	767	760: Cytochrome bc1 complex (Ubiquinol-cytochrome c reductase complex, complex III)<br>767: Cytochrome bc1 complex (Ubiquinol-cytochrome c reductase complex, complex III)
760	768	760: Cytochrome bc1 complex (Ubiquinol-cytochrome c reductase complex, complex III)<br>768: Cytochrome bc1 complex (Ubiquinol-cytochrome c reductase complex, complex III)
774	775	774: sulfite reductase complex (NADPH)<br>775: sulfite reductase complex (NADPH)
857	1118	857: U2 snRNP complex<br>857: SF3b complex<br>1118: SF3b complex
862	863	862: Cytochrome c oxidase (complex IV)<br>862: Cox14p/Cox1p/Mss51p complex<br>863: Cytochrome c oxidase (complex IV)
862	864	862: Cytochrome c oxidase (complex IV)<br>862: Cox14p/Cox1p/Mss51p complex<br>864: Cytochrome c oxidase (complex IV)
901	903	901: PAM complex<br>903: PAM complex
933	934	933: degradosome<br>934: degradosome
940	947	940: cytoplasmic ribosomal large subunit<br>947: cytoplasmic ribosomal large subunit
940	950	940: cytoplasmic ribosomal large subunit<br>950: cytoplasmic ribosomal large subunit
940	964	940: cytoplasmic ribosomal large subunit<br>964: cytoplasmic ribosomal large subunit
940	965	940: cytoplasmic ribosomal large subunit<br>965: cytoplasmic ribosomal large subunit
940	966	940: cytoplasmic ribosomal large subunit<br>966: cytoplasmic ribosomal large subunit
940	987	940: cytoplasmic ribosomal large subunit<br>987: cytoplasmic ribosomal large subunit
940	988	940: cytoplasmic ribosomal large subunit<br>988: cytoplasmic ribosomal large subunit
940	993	940: cytoplasmic ribosomal large subunit<br>993: cytoplasmic ribosomal large subunit
940	994	940: cytoplasmic ribosomal large subunit<br>994: cytoplasmic ribosomal large subunit
940	996	940: cytoplasmic ribosomal large subunit<br>996: cytoplasmic ribosomal large subunit
940	997	940: cytoplasmic ribosomal large subunit<br>997: cytoplasmic ribosomal large subunit
940	998	940: cytoplasmic ribosomal large subunit<br>998: cytoplasmic ribosomal large subunit
940	999	940: cytoplasmic ribosomal large subunit<br>999: cytoplasmic ribosomal large subunit
940	1000	940: cytoplasmic ribosomal large subunit<br>1000: cytoplasmic ribosomal large subunit
940	1001	940: cytoplasmic ribosomal large subunit<br>1001: cytoplasmic ribosomal large subunit
940	1003	940: cytoplasmic ribosomal large subunit<br>1003: cytoplasmic ribosomal large subunit
1183	1185	1183: Succinate dehydrogenase complex (complex II)<br>1185: Succinate dehydrogenase complex (complex II)
1328	1329	1328: LMA1 complex<br>1329: LMA1 complex
1372	1375	1372: cytoplasmic ribosomal small subunit<br>1375: cytoplasmic ribosomal small subunit
1372	1379	1372: cytoplasmic ribosomal small subunit<br>1379: cytoplasmic ribosomal small subunit
1372	1384	1372: cytoplasmic ribosomal small subunit<br>1384: cytoplasmic ribosomal small subunit
1372	1390	1372: cytoplasmic ribosomal small subunit<br>1390: cytoplasmic ribosomal small subunit
1372	1396	1372: cytoplasmic ribosomal small subunit<br>1396: cytoplasmic ribosomal small subunit
1372	1397	1372: cytoplasmic ribosomal small subunit<br>1397: cytoplasmic ribosomal small subunit
1372	1401	1372: cytoplasmic ribosomal small subunit<br>1401: cytoplasmic ribosomal small subunit
1372	1406	1372: cytoplasmic ribosomal small subunit<br>1406: cytoplasmic ribosomal small subunit
1372	1408	1372: cytoplasmic ribosomal small subunit<br>1408: cytoplasmic ribosomal small subunit
1372	1409	1372: cytoplasmic ribosomal small subunit<br>1409: cytoplasmic ribosomal small subunit
1372	1410	1372: cytoplasmic ribosomal small subunit<br>1410: cytoplasmic ribosomal small subunit
1372	1412	1372: cytoplasmic ribosomal small subunit<br>1412: cytoplasmic ribosomal small subunit
1372	1413	1372: cytoplasmic ribosomal small subunit<br>1413: cytoplasmic ribosomal small subunit
1372	1416	1372: cytoplasmic ribosomal small subunit<br>1416: cytoplasmic ribosomal small subunit
1372	1417	1372: cytoplasmic ribosomal small subunit<br>1417: cytoplasmic ribosomal small subunit
1431	1432	1431: glycosylphosphatidylinositol-N-acetylglucosaminyltransferase (GPI-GnT) complex<br>1432: glycosylphosphatidylinositol-N-acetylglucosaminyltransferase (GPI-GnT) complex
1431	1433	1431: glycosylphosphatidylinositol-N-acetylglucosaminyltransferase (GPI-GnT) complex<br>1433: glycosylphosphatidylinositol-N-acetylglucosaminyltransferase (GPI-GnT) complex
1431	1434	1431: glycosylphosphatidylinositol-N-acetylglucosaminyltransferase (GPI-GnT) complex<br>1434: glycosylphosphatidylinositol-N-acetylglucosaminyltransferase (GPI-GnT) complex
1507	1511	1507: mitochondrial outer membrane translocase complex<br>1511: mitochondrial outer membrane translocase complex
1566	1567	1566: HMC Complex<br>1567: HMC Complex
1583	1601	1583: mitochondrial ribosomal small subunit<br>1601: mitochondrial ribosomal small subunit
1583	1613	1583: mitochondrial ribosomal small subunit<br>1613: mitochondrial ribosomal. small subunit

Table A9: Frequency distribution of the assigned categories of additional proteins on BioGRID from PANTHER analysis, (a) Biological process (b) Cellular component (c) Molecular function. FQ shows the count for each matched category.

(a) 44 categories of biological process.

Category of biological process	FQ
cellular process	41
nitrogen compound metabolic process	27
biosynthetic process	17
phosphate-containing compound metabolic process	15
nucleobase-containing compound metabolic process	12
respiratory electron transport chain	11
cellular component biogenesis	11
oxidative phosphorylation	10
catabolic process	8
organelle organization	7
mitochondrion organization	5
rRNA metabolic process	5
regulation of biological process	4
translation	4
cellular protein modification process	4
cation transport	4
protein targeting	3
protein localization	3
protein complex assembly	3
proteolysis	3
RNA splicing, via transesterification reactions	3
mRNA splicing, via spliceosome	3
cellular amino acid biosynthetic process	2
RNA catabolic process	2
mitochondrial transport	2
tRNA metabolic process	2
mitochondrial transport	2
tRNA metabolic process	2
tricarboxylic acid cycle	1
protein folding	1
nucleobase-containing compound transport	1
response to stress	1
protein metabolic process	1
RNA localization	1
intracellular protein transport	1
cellular amino acid catabolic process	1
protein phosphorylation	1
regulation of phosphate metabolic process	1
regulation of translation	1
response to stimulus	1
cellular component movement	1
cytoskeleton organization	1
transport	1
ferredoxin metabolic process	1

(b) 17 categories of cellular component.

Category of cellular component	FQ
ribosome	26
cytosol	25
cytoplasm	25
organelle	21
protein complex	18
mitochondrial inner membrane	11
mitochondrion	8
nucleus	7
intracellular	3
endoplasmic reticulum	2
ribonucleoprotein complex	2
proton-transporting ATP synthase complex	1
integral to membrane	1
plasma membrane	1
nuclear outer membrane-endoplasmic reticulum membrane network	1
vacuole	1
cytoskeleton	1

(c) 26 categories of molecular function.

Category of molucular function	FQ
structural constituent of ribosome	20
oxidoreductase activity	13
binding	7
protein binding	6
RNA binding	5
cation transmembrane transporter activity	5
transmembrane transporter activity	3
nucleotide binding	3
pyrophosphatase activity	3
hydrogen ion transmembrane transporter activity	3
kinase activity	2
enzyme regulator activity	2
mRNA binding	1
structural constituent of ribosome	1
metallopeptidase activity	1
acetyltransferase activity	1
microtubule motor activity	1
GTPase activity	1
signal transducer activity	1
exoribonuclease activity	1
transferase activity, transferring glycosyl groups	1
small GTPase regulator activity	1
peptidase inhibitor activity	1
RNA helicase activity	1
hydrolase activity, acting on ester bonds	1
guanyl-nucleotide exchange factor activity	1

Table A10 : The additional protein pairs for supporting CYC2008 protein complex and the protein complexes that are not fully covered with BioGRID.

(a) The additional protein pairs for supporting CYC2008 complexes on WI-PHI.

Systematic name of the protein pair	
ADA2	CND1
ADA2	RTG2
ADA2	SUS1
SBH2	SSH1
SBH2	SSS1
SSS1	SBH1
MSS1	MTO1
ELC1	CUL3
EST1	EST3
MDM10	SAM35
CDC28	CLB6
INH1	STF2
INH1	ATP19
SNU13	SMB1
SNU13	PRP18
TAF14	EAF6
HOP1	ZIP1
HOP1	ZIP2
RSP5	BUL2
HTB1	CHZ1
HTB1	HTZ1
IMG1	MRPL11
DYN1	DYN2
DYN1	DYN3
ALG13	ALG14
LPD1	GCV1
LPD1	GCV2
LPD1	GCV3
LPD1	PKP1
LPD1	PTC5
USA1	DER1
MGR1	YME1
ARG3	CAR1
ARG2	ARG5,6
COB	QCR10
FET3	FTR1
GTR2	LTV1
CSM1	HRR25
RSE1	YSF3
COX1	COX12
COX1	COX13
MGE1	PAM17
RMD5	YDL176W
BI4	NAM2
DSS1	SUV3
BUD32	PCC1
RPL10	RPL20B
RPL10	RPL21B
RPL10	RPL22A
RPL10	RPL22B
RPL10	RPL24B
RPL10	RPL29
RPL10	RPL32
RPL10	RPL34A
RPL10	RPL37B
RPL10	RPL39
RPL10	RPL40A
RPL10	RPL40B
RPL10	RPL41A
RPL10	RPL41B
SEC11	SPC2
SEC11	SPC3
BIR1	SLI15
SDH1	SDH3
SDH1	SDH4
DUG1	DUG2
PEP3	VPS3
PBI2	YLR042C
BBP1	NBP1
ASC1	RPS12
ASC1	RPS17A
ASC1	RPS19A
ASC1	RPS19B
ASC1	RPS23A
ASC1	RPS23B
ASC1	RPS24B
ASC1	RPS25A
ASC1	RPS27A
ASC1	RPS27B
ASC1	RPS28A
ASC1	RPS29B
ASC1	RPS30A
ASC1	RPS30B
ASC1	RPS31
ASC1	RPS8B
ERT1	GPI1
ERT1	GPI15
ERT1	GPI19
ERT1	GPI2
GSC2	SMK1
HSC82	SRO9
MRP1	PPE1
MRP1	YMR31

(b) The list of the protein complexes that are not fully covered with WI-PHI.

Name of the protein complex
Ada2p/Gcn5p/Ada3 transcription activator complex
SAGA complex
SLIK (SAGA-like) complex
SAGA complex
Mks1p/Rtg2p complex
Ssh1p translocon complex
Sec61p translocon complex
Mto1p/Mss1p complex
nucleotide-excision repair factor 4 complex
Ela1p/Elc1p complex
Cul3p-RING ubiquitin ligase complex
Telomerase
mitochondrial sorting and assembly machinery complex
Mdm12p/Mmm1p/Mdm10p complex
Cdc28p/Clb5p complex
Cdc28p/Cln2p complex
Cdc28p/Clb1p complex
Cdc28p/Clb6p complex
Cdc28p/Clb3p complex
Cdc28p/Cln3p complex
Cdc28p/Clb4p complex
Cdc28p/Clb2p complex
mitochondrial ATPase inhibitor complex
F0/F1 ATP synthase (complex V)
box C/D snoRNP complex
U4/U6 x U5 tri-snRNP complex
commitment complex
U5 snRNP complex
U2 snRNP complex
U1 snRNP complex
Ino80p complex
SWI/SNF complex
transcription factor TFIIF complex
NuA3 histone acetyltransferase complex
NuA4 histone acetyltransferase complex
Synaptonemal complex (SC)
Rsp5p/Bul1 ubiquitin ligase complex
Rsp5p/Bul2 ubiquitin ligase complex
Nucleosomal protein complex
Chz1p/Htz1p/Htb1p complex
mitochondrial ribosomal large subunit
cytoplasmic dynein complex
UDP-N-acetylglucosamine transferase complex
mitochondrial oxoglutarate dehydrogenase complex
glycine cleavage complex
mitochondrial pyruvate dehydrogenase complex
ubiquitin ligase ERAD-L complex
i-AAA complex
Car1p/Arg3p complex
Arg2p/Arg5,6p complex
Cytochrome bc1 complex (Ubiquinol-cytochrome c reductase complex, complex III)
Permease-Oxidase Complex
EGO complex
GSE Complex
Csm1p/Lrs4p complex
monopolin complex
U2 snRNP complex
SF3b complex
Cytochrome c oxidase (complex IV)
Cox14p/Cox1p/Mss51p complex
PAM complex
FBP degradation complex
Bi4p/Nam2p complex
degradosome
EKC/KEOPS protein complex
cytoplasmic ribosomal large subunit
signal peptidase complex
Sli15p/Bir1p complex
Aurora B-INCENP protein kinase complex
Succinate dehydrogenase complex (complex II)
GSH degradosomal complex
CORVET complex
HOPS complex
LMA1 complex
Mps2p/Bbp1p complex
cytoplasmic ribosomal small subunit
glycosylphosphatidylinositol-N-acetylglucosaminyltransferase (GPI-GnT) complex
1,3-beta-glucan synthase complex (Gsc2p/Smk1p)
HMC Complex
mitochondrial ribosomal small subunit

Table A11: The additional protein pairs and the included complexes on WI-PHI. These proteins correspond to the protein pairs described in Table A10(a). For example, The name of the gene encording the protein numbering 14 is ADA2 which is a subunit of the following complexes, Ada2p/Gcn5p/Ada3 transcription activator complex, SAGA complex and SLIK (SAGA-like) complex.

14	1273	14: Ada2p/Gcn5p/Ada3 transcription activator complex<br>14: SAGA complex<br>14: SLIK (SAGA-like) complex<br>1273: SAGA complex<br>1273: SLIK (SAGA-like) complex
14	62	14: Ada2p/Gcn5p/Ada3 transcription activator complex<br>14: SAGA complex<br>14: SLIK (SAGA-like) complex<br>62: Mks1p/Rtg2p complex<br>62: SLIK (SAGA-like) complex
14	1281	14: Ada2p/Gcn5p/Ada3 transcription activator complex<br>14: SAGA complex<br>14: SLIK (SAGA-like) complex<br>1281: SAGA complex
45	46	45: Ssh1p translocon complex<br>46: Ssh1p translocon complex
45	47	45: Ssh1p translocon complex<br>47: Ssh1p translocon complex<br>47: Sec61p translocon complex
47	1440	47: Ssh1p translocon complex<br>47: Sec61p translocon complex<br>1440: Sec61p translocon complex
57	58	57: Mto1p/Mss1p complex<br>58: Mto1p/Mss1p complex
127	1024	127: nucleotide-excision repair factor 4 complex<br>127: Ela1p/Elc1p complex<br>127: Cul3p-RING ubiquitin ligase complex<br>1024: Cul3p-RING ubiquitin ligase complex
130	132	130: Telomerase<br>132: Telomerase
150	151	150: mitochondrial sorting and assembly machinery complex<br>150: Mdm12p/Mmm1p/Mdm10p complex<br>151: mitochondrial sorting and assembly machinery complex
169	787	169: Cdc28p/Clb5p complex<br>169: Cdc28p/Cln2p complex<br>169: Cdc28p/Clb1p complex<br>169: Cdc28p/Clb6p complex<br>169: Cdc28p/Cln1p complex<br>169: Cdc28p/Clb3p complex<br>169: Cdc28p/Cln3p complex<br>169: Cdc28p/Clb4p complex<br>169: Cdc28p/Clb2p complex<br>787: Cdc28p/Clb6p complex
218	220	218: mitochondrial ATPase inhibitor complex<br>218: F0/F1 ATP synthase (complex V)<br>220: mitochondrial ATPase inhibitor complex
218	516	218: mitochondrial ATPase inhibitor complex<br>218: F0/F1 ATP synthase (complex V)<br>516: F0/F1 ATP synthase (complex V)
229	235	229: box C/D snoRNP complex<br>229: U4/U6 x U5 tri-snRNP complex<br>235: commitment complex<br>235: U5 snRNP complex<br>235: U4/U6 x U5 tri-snRNP complex<br>235: U2 snRNP complex<br>235: U1 snRNP complex
229	699	229: box C/D snoRNP complex<br>229: U4/U6 x U5 tri-snRNP complex<br>699: U4/U6 x U5 tri-snRNP complex
331	640	331: Ino80p complex<br>331: SWI/SNF complex<br>331: transcription factor TFIIF complex<br>331: NuA3 histone acetyltransferase complex<br>331: transcription factor TFIID complex<br>640: NuA3 histone acetyltransferase complex<br>640: NuA4 histone acetyltransferase complex
431	433	431: Synaptonemal complex (SC)<br>433: Synaptonemal complex (SC)
431	434	431: Synaptonemal complex (SC)<br>434: Synaptonemal complex (SC)
439	930	439: Rsp5p/Bul1 ubiquitin ligase complex<br>439: Rsp5p/Bul2 ubiquitin ligase complex<br>930: Rsp5p/Bul2 ubiquitin ligase complex
493	750	493: Nucleosomal protein complex<br>493: Chz1p/Htz1p/Htb1p complex<br>750: Chz1p/Htz1p/Htb1p complex
493	751	493: Nucleosomal protein complex<br>493: Chz1p/Htz1p/Htb1p complex<br>751: Chz1p/Htz1p/Htb1p complex
527	535	527: mitochondrial ribosomal large subunit<br>535: mitochondrial ribosomal large subunit
617	618	617: cytoplasmic dynein complex<br>618: cytoplasmic dynein complex
617	619	617: cytoplasmic dynein complex<br>619: cytoplasmic dynein complex
621	622	621: UDP-N-acetylglucosamine transferase complex<br>622: UDP-N-acetylglucosamine transferase complex
656	889	656: mitochondrial oxoglutarate dehydrogenase complex<br>656: glycine cleavage complex<br>656: mitochondrial pyruvate dehydrogenase complex<br>889: glycine cleavage complex
656	890	656: mitochondrial oxoglutarate dehydrogenase complex<br>656: glycine cleavage complex<br>656: mitochondrial pyruvate dehydrogenase complex<br>890: glycine cleavage complex
656	891	656: mitochondrial oxoglutarate dehydrogenase complex<br>656: glycine cleavage complex<br>656: mitochondrial pyruvate dehydrogenase complex<br>891: glycine cleavage complex
656	1345	656: mitochondrial oxoglutarate dehydrogenase complex<br>656: glycine cleavage complex<br>656: mitochondrial pyruvate dehydrogenase complex<br>1345: mitochondrial pyruvate dehydrogenase complex
656	1346	656: mitochondrial oxoglutarate dehydrogenase complex<br>656: glycine cleavage complex<br>656: mitochondrial pyruvate dehydrogenase complex<br>1346: mitochondrial pyruvate dehydrogenase complex
670	671	670: ubiquitin ligase ERAD-L complex<br>671: ubiquitin ligase ERAD-L complex
708	709	708: i-AAA complex<br>709: i-AAA complex
713	714	713: Car1p/Arg3p complex<br>714: Car1p/Arg3p complex
717	718	717: Arg2p/Arg5,6p complex<br>718: Arg2p/Arg5,6p complex
760	763	760: Cytochrome bc1 complex (Ubiquinol-cytochrome c reductase complex, complex III)<br>763: Cytochrome bc1 complex (Ubiquinol-cytochrome c reductase complex, complex III)
780	781	780: Permease-Oxidase Complex<br>781: Permease-Oxidase Complex
795	1216	795: EGO complex<br>795: GSE Complex<br>1216: GSE Complex
836	1618	836: Csm1p/Lrs4p complex<br>836: monopolin complex<br>1618: monopolin complex
857	1118	857: U2 snRNP complex<br>857: SF3b complex<br>1118: SF3b complex
862	863	862: Cytochrome c oxidase (complex IV)<br>862: Cox14p/Cox1p/Mss51p complex<br>863: Cytochrome c oxidase (complex IV)
862	864	862: Cytochrome c oxidase (complex IV)<br>862: Cox14p/Cox1p/Mss51p complex<br>864: Cytochrome c oxidase (complex IV)
901	903	901: PAM complex<br>903: PAM complex
917	924	917: FBP degradation complex<br>924: FBP degradation complex
931	932	931: Bi4p/Nam2p complex<br>932: Bi4p/Nam2p complex
933	934	933: degradosome<br>934: degradosome
935	939	935: EKC/KEOPS protein complex<br>939: EKC/KEOPS protein complex
940	962	940: cytoplasmic ribosomal large subunit<br>962: cytoplasmic ribosomal large subunit
940	964	940: cytoplasmic ribosomal large subunit<br>964: cytoplasmic ribosomal large subunit
940	965	940: cytoplasmic ribosomal large subunit<br>965: cytoplasmic ribosomal large subunit
940	966	940: cytoplasmic ribosomal large subunit<br>966: cytoplasmic ribosomal large subunit
940	970	940: cytoplasmic ribosomal large subunit<br>970: cytoplasmic ribosomal large subunit
940	977	940: cytoplasmic ribosomal large subunit<br>977: cytoplasmic ribosomal large subunit
940	984	940: cytoplasmic ribosomal large subunit<br>984: cytoplasmic ribosomal large subunit
940	987	940: cytoplasmic ribosomal large subunit<br>987: cytoplasmic ribosomal large subunit
940	994	940: cytoplasmic ribosomal large subunit<br>994: cytoplasmic ribosomal large subunit
940	996	940: cytoplasmic ribosomal large subunit
940	997	940: cytoplasmic ribosomal large subunit<br>997: cytoplasmic ribosomal large subunit
940	998	940: cytoplasmic ribosomal large subunit<br>998: cytoplasmic ribosomal large subunit
940	999	940: cytoplasmic ribosomal large subunit<br>999: cytoplasmic ribosomal large subunit
940	1000	940: cytoplasmic ribosomal large subunit<br>1000: cytoplasmic ribosomal large subunit
1095	1097	1095: signal peptidase complex<br>1097: signal peptidase complex
1095	1098	1095: signal peptidase complex<br>1098: signal peptidase complex
1139	1140	1139: Sli15p/Bir1p complex<br>1140: Sli15p/Bir1p complex<br>1140: Aurora B-INCENP protein kinase complex
1183	1185	1183: Succinate dehydrogenase complex (complex II)<br>1185: Succinate dehydrogenase complex (complex II)
1183	1186	1183: Succinate dehydrogenase complex (complex II)<br>1186: Succinate dehydrogenase complex (complex II)
1234	1235	1234: GSH degradosomal complex<br>1235: GSH degradosomal complex
1253	1255	1253: CORVET complex<br>1253: HOPS complex<br>1255: CORVET complex
1328	1329	1328: LMA1 complex<br>1329: LMA1 complex
1347	1349	1347: Mps2p/Bbp1p complex<br>1349: Mps2p/Bbp1p complex
1372	1379	1372: cytoplasmic ribosomal small subunit<br>1379: cytoplasmic ribosomal small subunit
1372	1386	1372: cytoplasmic ribosomal small subunit<br>1386: cytoplasmic ribosomal small subunit
1372	1390	1372: cytoplasmic ribosomal small subunit<br>1390: cytoplasmic ribosomal small subunit
1372	1391	1372: cytoplasmic ribosomal small subunit<br>1391: cytoplasmic ribosomal small subunit
1372	1400	1372: cytoplasmic ribosomal small subunit<br>1400: cytoplasmic ribosomal small subunit
1372	1401	1372: cytoplasmic ribosomal small subunit<br>1401: cytoplasmic ribosomal small subunit
1372	1403	1372: cytoplasmic ribosomal small subunit<br>1403: cytoplasmic ribosomal small subunit
1372	1404	1372: cytoplasmic ribosomal small subunit<br>1404: cytoplasmic ribosomal small subunit
1372	1408	1372: cytoplasmic ribosomal small subunit<br>1408: cytoplasmic ribosomal small subunit
1372	1409	1372: cytoplasmic ribosomal small subunit<br>1409: cytoplasmic ribosomal small subunit
1372	1410	1372: cytoplasmic ribosomal small subunit<br>1410: cytoplasmic ribosomal small subunit
1372	1413	1372: cytoplasmic ribosomal small subunit<br>1413: cytoplasmic ribosomal small subunit
1372	1415	1372: cytoplasmic ribosomal small subunit<br>1415: cytoplasmic ribosomal small subunit
1372	1416	1372: cytoplasmic ribosomal small subunit<br>1416: cytoplasmic ribosomal small subunit
1372	1417	1372: cytoplasmic ribosomal small subunit<br>1417: cytoplasmic ribosomal small subunit
1372	1426	1372: cytoplasmic ribosomal small subunit<br>1426: cytoplasmic ribosomal small subunit
1431	1432	1431: glycosylphosphatidylinositol-N-acetylglucosaminyltransferase (GPI-GnT) complex<br>1432: glycosylphosphatidylinositol-N-acetylglucosaminyltransferase (GPI-GnT) complex
1431	1433	1431: glycosylphosphatidylinositol-N-acetylglucosaminyltransferase (GPI-GnT) complex<br>1433: glycosylphosphatidylinositol-N-acetylglucosaminyltransferase (GPI-GnT) complex
1431	1434	1431: glycosylphosphatidylinositol-N-acetylglucosaminyltransferase (GPI-GnT) complex<br>1434: glycosylphosphatidylinositol-N-acetylglucosaminyltransferase (GPI-GnT) complex
1431	1435	1431: glycosylphosphatidylinositol-N-acetylglucosaminyltransferase (GPI-GnT) complex<br>1435: glycosylphosphatidylinositol-N-acetylglucosaminyltransferase (GPI-GnT) complex
1458	1459	1458: 1,3-beta-glucan synthase complex (Gsc2p/Smk1p)<br>1459: 1,3-beta-glucan synthase complex (Gsc2p/Smk1p)
1566	1567	1566: HMC Complex<br>1567: HMC Complex
1583	1601	1583: mitochondrial ribosomal small subunit<br>1601: mitochondrial ribosomal small subunit
1583	1613	1583: mitochondrial ribosomal small subunit<br>1613: mitochondrial ribosomal small subunit

Table A12: Frequency distribution of the assigned categories of additional proteins on WI-PHI from PANTHER analysis, (a) Biological process (b) Cellular component (c) Molecular function. FQ shows the count for each matched category.

(a) 78 categories of biological process.

Category of biological process	FQ
cellular process	71
nitrogen compound metabolic process	40
biosynthetic process	28
phosphate-containing compound metabolic process	19
catabolic process	19
cellular component biogenesis	13
nucleobase-containing compound metabolic process	12
organelle organization	12
regulation of biological process	11
cellular protein modification process	10
translation	10
respiratory electron transport chain	8
oxidative phosphorylation	7
proteolysis	7
protein localization	6
protein targeting	6
cation transport	5
response to stimulus	4
regulation of transcription from RNA polymerase II promoter	4
mRNA splicing, via spliceosome	4
chromatin organization	4
RNA splicing, via transesterification reactions	4
rRNA metabolic process	4
RNA catabolic process	4
RNA localization	3
cellular amino acid catabolic process	3
tRNA metabolic process	3
response to stress	3
mitochondrion organization	3
cellular amino acid biosynthetic process	3
protein acetylation	2
mitochondrial transport	2
tricarboxylic acid cycle	2
chromatin remodeling	2
protein complex assembly	2
cellular component morphogenesis	2
homeostatic process	2
protein metabolic process	2
nucleobase-containing compound transport	2
nuclear transport	2
protein phosphorylation	2
transport	1
cellular component organization	1
transcription from RNA polymerase II promoter	1
regulation of cellular amino acid metabolic process	1
cellular amino acid metabolic process	1
cell surface receptor signaling pathway	1
intracellular protein transport	1
endocytosis	1
cytoskeleton organization	1
single-multicellular organism process	1
protein folding	1
cellular component movement	1
cell cycle	1
G-protein coupled receptor signaling pathway	1
MAPK cascade	1
response to external stimulus	1
cell proliferation	1
regulation of nucleobase-containing compound metabolic process	1
mitochondrial translation	1
DNA replication	1
tRNA aminoacylation for protein translation	1
regulation of translation	1
regulation of phosphate metabolic process	1
mRNA polyadenylation	1
developmental process	1
vesicle-mediated transport	1
signal transduction	1
lipid transport	1
intracellular signal transduction	1
ferredoxin metabolic process	1
regulation of gene expression, epigenetic	1
transcription elongation from RNA polymerase II promoter	1
anion transport	1
regulation of cell cycle	1
transcription, DNA-dependent	1
response to endogenous stimulus	1
regulation of catalytic activity	1

(b) 21 categories of cellular component.

Category of cellular component	FQ
cytoplasm	38
protein complex	30
cytosol	26
ribosome	24
organelle	22
nucleus	17
intracellular	11
endoplasmic reticulum	8
mitochondrial inner membrane	8
mitochondrion	7
nuclear outer membrane-endoplasmic reticulum membrane network	6
ribonucleoprotein complex	5
vacuole	5
membrane	4
endosome	3
nucleoplasm	3
plasma membrane	3
cytoskeleton	2
lysosome	1
nuclear chromosome	1
proton-transporting ATP synthase complex	1

(c) 42 categories of molecular function.

Category of molecular function	FQ
structural constituent of ribosome	17
oxidoreductase activity	12
protein binding	12
binding	7
pyrophosphatase activity	6
RNA binding	5
protein kinase activity	5
nucleotide binding	4
cation transmembrane transporter activity	4
chromatin binding	4
transmembrane transporter activity	3
GTPase activity	3
peptidase activity	3
transcription cofactor activity	3
ubiquitin-protein ligase activity	3
acetyltransferase activity	3
hydrogen ion transmembrane transporter activity	3
guanyl-nucleotide exchange factor activity	2
small GTPase regulator activity	2
kinase activity	2
microtubule motor activity	2
signal transducer activity	2
metallopeptidase activity	2
enzyme regulator activity	2
DNA binding	2
receptor activity	1
exoribonuclease activity	1
hydrolase activity, acting on ester bonds	1
sequence-specific DNA binding transcription factor activity	1
aminoacyl-tRNA ligase activity	1
transferase activity, transferring glycosyl groups	1
catalytic activity	1
kinase inhibitor activity	1
phosphoprotein phosphatase activity	1
kinase activator activity	1
RNA-directed DNA polymerase activity	1
transferase activity	1
hydrolase activity	1
nuclease activity	1
nucleotidyltransferase activity	1
RNA helicase activity	1
peptidase inhibitor activity	1

Table A13 : The additional protein pairs for supporting CYC2008 protein complex and the protein complexes that are not fully covered with iRefIndex.

(a) The additional protein pairs for supporting CYC2008 complexes on iRefIndex.

Systematic name of the protein pair	
MET28	MET4
STE18	STE4
KEL1	KEL2
DPB4	DPB2
DPB4	DPB3
DBP4	POL2
MST27	MST28
CBF1	MET28
MET28	MET31
PRP11	PRP21
PRP11	PRP9
KEL1	LTE1
MSS1	MTO1
GIP1	GLC7
GLC7	BNI4
GLC7	REG1
GLC7	BUD14
GLC7	SDS22
GLC7	GAC1
CSG2	CSH1
DDC1	MEC3
DDC1	RAD17
ABF1	ELC1
TRM11	TRM112
DBF2	MOB1
SIT4	RRD1
BUR6	NCB2
FRS1	FRS2
PPH3	PSY4
TUB1	TUB2
IME1	UME6
SIP1	SNF1
PET3	PMT5
SEC9	SRO7
PSR1	WHI2
BUR2	SGV1
POL3	POL31
POL3	POL32
GAL80	GAL3
HOP1	RED1
HOP1	ZIP1
HOP1	ZIP2
GET1	GET2
GET1	GET3
RVS161	RVS167
ARG80	ARG81
ARG80	ARG82
ARG80	MCM1
HPR1	MFT1
HPR1	THO2
HPR1	THP2
SCC2	SCC4
PHO85	PCL10
PHO85	PCL2
CYR1	SRV2
HSE1	VPS27
NSP1	NUP82
ARG2	ARG5,6
SPT15	MOT1
CDC7	DBF4
GYL1	GYP5
BI3	MRS1
CPA1	CPA2
BI4	NAM2
STE18	GPA1
BIR1	SLI15
SLI15	IPL1
RAD1	RAD10
RAD1	RAD14
CSM3	MRC1
CSM3	TOF1
CHC1	CLC1
DUG1	DUG2
RAD23	DNG1
DEF1	RAD26
MMS4	MUS81
PBI2	TRX1
SUP35	SUP45
ERI1	GPI1
ERI1	GPI15
GSC2	SMK1
CCS1	SOD1
SPR101	SRP102
TRP2	TRP3
NEM1	SPO7

(b) The list of the protein complexes that are not fully covered with iRefIndex.

Name of the protein complex
Cbf1p/Met4p/Met28p complex
Met4p/Met28p/Met31p complex
Met4p/Met28p/Met32p complex
Cdc24p/Far1p/Gbetagamma protein complex
heterodimeric G-protein complex
Kel1p/Kel2p complex
Kel1p/Kel2p/Lte1p complex
chromatin accessibility complex
DNA polymerase epsilon complex
Mst27p/Mst28p complex
Prp9p/Prp11p/Prp21p complex(SF3a complex)
U2 snRNP complex
Mto1p/Mss1p complex
Gip1p/Glc7p complex
mRNA cleavage and polyadenylation
specificity factor complex
Reg2p/Glc7p complex
Bni4p/Glc7p complex
Reg1p/Glc7p complex
Bud14p/Glc7p complex
Sds22p/Glc7p complex
Gac1p/Glc7p complex
Sur1p/Csg2p complex
Csh1p/Csg2p complex
Rad17p/Ddc1p/Mec3p complex
nucleotide-excision repair factor 4 complex
Ela1p/Elc1p complex
Cul3p-RING ubiquitin ligase complex
adoMet-dependent tRNA methyltransferase (Mtase) Complex
Mob1p/Dbf2p complex
Sap190p/Sit4p complex
Sap155p/Sit4p complex
Tap42p/Sit4p/Rrd1p complex
Sap185p/Sit4p complex
NC2 complex
Phenylalaninine-tRNA-ligase
histone H2A phosphatase complex
Tubulins
Ume6p/Ime1p complex
Rpd3L complex
Snf1p/Snf4p/Sip1p complex
Snf1p/Snf4p/Gal83p complex
Snf1p/Snf4p/Sip2p complex
Protein O-mannosyltransferase(Pmt3p/Pmt5p)
SNARE complex
Sro1p/Sec4p/Sec9p complex
Psr1p/Whi2p complex
Sgv1p/Bur2p complex
DNA polymerase delta complex
GAL4p/GAL80p complex
GAL3p/GAL80p complex
Synaptonemal complex (SC)
GET complex
Rvs161p/Rvs167p complex
ARGR complex
Cdc73p/Paf1p complex
THO complex
cohesin loading factor complex
Pho85p/Pcl8p complex
Pho85p/Pho80p complex
Pho85p/Pcl10p complex
Pho85p/Pcl1p complex
Pho85p/Pcl2p complex
Pho85p/Pcl10p complex
adenylyl cyclase complex
Hse1p/Vps27p complex
Nsp1p/Nup82p complex
Nsp1p complex
Nup82 nuclear pore subcomplex
Arg2p/Arg5,6p complex
transcription factor TFIIIB complex
transcription factor TFIID complex
Mot1p complex
Cdc7p/Dbf4p complex
Gyl1p/Gyp5p complex
Bi3 ribonucleoprotein complex
carbamoyl-phosphate synthase complex
Bi4p/Nam2p complex
Cdc24p/Far1p/Gbetagamma protein complex
heterodimeric G-protein complex
Sli15p/Bir1p complex
Aurora B-INCENP protein kinase complex
nucleotide-excision repair factor 1 complex
Tof1p/Mrc1p/Csm3p complex
Clathrin
GSH degradosomal complex
nucleotide-excision repair factor 2 complex
Png1p/Rad23p complex
Def1p/Rad26p complex
Holliday junction resolvase complex
LMA1 complex
translation release factor complex
glycosylphosphatidylinositol-N-acetylglucosaminyltransferase (GPI-GnT) complex
1,3-beta-glucan synthase complex (Gsc2p/Smk1p)
Sod1p/Ccs1p complex
signal recognition particle receptor complex
anthranilate synthase complex
Nem1p/Spo7p complex

Table A14 : The additional protein pairs and the included complexes on iRefIndex. These proteins correspond to the protein pairs described in Table A13(a). For example, The name of the gene encording the protein numbering 31 is MET28 which is a subunit of the following complexes, Cbf1p/Met4p/Met28p complex, Met4p/Met28p/Met31p complex and Met4p/Met28p/Met32p complex.

31	32	31: Cbf1p/Met4p/Met28p complex<br>31: Met4p/Met28p/Met31p complex<br>31: Met4p/Met28p/Met32p complex<br>32: Cbf1p/Met4p/Met28p complex<br>32: Met4p/Met28p/Met31p complex<br>32: Met4p/Met28p/Met32p complex
1084	1085	1084: Cdc24p/Far1p/Gbetagamma protein complex<br>1084: heterodimeric G-protein complex<br>1084: heterotrimeric G-protein complex<br>1085: Cdc24p/Far1p/Gbetagamma protein complex<br>1085: heterodimeric G-protein complex<br>1085: heterotrimeric G-protein complex
48	49	48: Kel1p/Kel2p complex<br>48: Kel1p/Kel2p/Lte1p complex<br>49: Kel1p/Kel2p complex<br>49: Kel1p/Kel2p/Lte1p complex
20	405	20: chromatin accessibility complex<br>20: DNA polymerase epsilon complex<br>405: DNA polymerase epsilon complex
20	406	20: chromatin accessibility complex<br>20: DNA polymerase epsilon complex<br>406: DNA polymerase epsilon complex
20	407	20: chromatin accessibility complex<br>20: DNA polymerase epsilon complex<br>407: DNA polymerase epsilon complex
23	24	23: Mst27p/Mst28p complex<br>24: Mst27p/Mst28p complex
30	31	30: Cbf1p/Met4p/Met28p complex<br>31: Cbf1p/Met4p/Met28p complex<br>31: Met4p/Met28p/Met31p complex<br>31: Met4p/Met28p/Met32p complex
31	36	31: Cbf1p/Met4p/Met28p complex<br>31: Met4p/Met28p/Met31p complex<br>31: Met4p/Met28p/Met32p complex<br>36: Met4p/Met28p/Met31p complex
33	34	33: Prp9p/Prp11p/Prp21p complex(SF3a complex)<br>33: U2 snRNP complex<br>34: Prp9p/Prp11p/Prp21p complex(SF3a complex)<br>34: U2 snRNP complex
33	35	33: Prp9p/Prp11p/Prp21p complex(SF3a complex)<br>33: U2 snRNP complex<br>35: Prp9p/Prp11p/Prp21p complex(SF3a complex)<br>35: U2 snRNP complex
48	486	48: Kel1p/Kel2p complex<br>48: Kel1p/Kel2p/Lte1p complex<br>486: Kel1p/Kel2p/Lte1p complex
57	58	57: Mto1p/Mss1p complex<br>58: Mto1p/Mss1p complex
81	82	81: Gip1p/Glc7p complex<br>82: Gip1p/Glc7p complex<br>82: mRNA cleavage and polyadenylation specificity factor complex<br>82: Reg2p/Glc7p complex<br>82: Bni4p/Glc7p complex<br>82: Reg1p/Glc7p complex<br>82: Bud14p/Glc7p complex<br>82: Sds22p/Glc7p complex<br>82: Gac1p/Glc7p complex
82	808	82: Gip1p/Glc7p complex<br>82: mRNA cleavage and polyadenylation specificity factor complex<br>82: Reg2p/Glc7p complex<br>82: Bni4p/Glc7p complex<br>82: Reg1p/Glc7p complex<br>82: Bud14p/Glc7p complex<br>82: Sds22p/Glc7p complex<br>82: Gac1p/Glc7p complex<br>808: Bni4p/Glc7p complex
82	927	82: Gip1p/Glc7p complex<br>82: mRNA cleavage and polyadenylation specificity factor complex<br>82: Reg2p/Glc7p complex<br>82: Bni4p/Glc7p complex<br>82: Reg1p/Glc7p complex<br>82: Bud14p/Glc7p complex<br>82: Sds22p/Glc7p complex<br>82: Gac1p/Glc7p complex<br>927: Reg1p/Glc7p complex
82	1196	82:Gip1p/Glc7p complex<br>82: mRNA cleavage and polyadenylation specificity factor complex<br>82: Reg2p/Glc7p complex<br>82: Bni4p/Glc7p complex<br>82: Reg1p/Glc7p complex<br>82: Bud14p/Glc7p complex<br>82: Sds22p/Glc7p complex<br>82: Gac1p/Glc7p complex<br>1196: Bud14p/Glc7p complex
82	1486	82: Gip1p/Glc7p complex<br>82: mRNA cleavage and polyadenylation specificity factor complex<br>82: Reg2p/Glc7p complex<br>82: Bni4p/Glc7p complex<br>82: Reg1p/Glc7p complex<br>82: Bud14p/Glc7p complex<br>82: Sds22p/Glc7p complex<br>82: Gac1p/Glc7p complex<br>1486: Sds22p/Glc7p complex
82	1574	82: Gip1p/Glc7p complex<br>82: mRNA cleavage and polyadenylation specificity factor complex<br>82: Reg2p/Glc7p complex<br>82: Bni4p/Glc7p complex<br>82: Reg1p/Glc7p complex<br>82: Bud14p/Glc7p complex<br>82: Sds22p/Glc7p complex<br>82: Gac1p/Glc7p complex<br>1574: Gac1p/Glc7p complex
83	1088	83: Sur1p/Csg2p complex<br>83: Csh1p/Csg2p complex<br>1088: Csh1p/Csg2p complex
113	114	113: Rad17p/Ddc1p/Mec3p complex<br>114: Rad17p/Ddc1p/Mec3p complex
113	115	113: Rad17p/Ddc1p/Mec3p complex<br>115: Rad17p/Ddc1p/Mec3p complex
126	127	126: nucleotide-excision repair factor 4 complex<br>127: nucleotide-excision repair factor 4 complex<br>127: Ela1p/Elc1p complex<br>127: Cul3p-RING ubiquitin ligase complex
133	134	133: adoMet-dependent tRNA methyltransferase (Mtase) Complex<br>134: adoMet-dependent tRNA methyltransferase (Mtase) Complex
186	187	186: Mob1p/Dbf2p complex6<br>187: Mob1p/Dbf2p complex
202	1189	202: Sap190p/Sit4p complex<br>202: Sap155p/Sit4p complex<br>202: Tap42p/Sit4p/Rrd1p complex<br>202: Sap185p/Sit4p complex<br>1189: Tap42p/Sit4p/Rrd1p complex
203	204	203: NC2 complex<br>204: NC2 complex
207	208	207: Phenylalaninine-tRNA-ligase<br>208: Phenylalaninine-tRNA-ligase
267	269	267: histone H2A phosphatase complex<br>269: histone H2A phosphatase complex
272	273	272: Tubulins<br>273: Tubulins
294	295	294: Ume6p/Ime1p complex<br>295: Ume6p/Ime1p complex<br>295: Rpd3L complex
306	307	306: Snf1p/Snf4p/Sip1p complex<br>307: Snf1p/Snf4p/Sip1p complex<br>307: Snf1p/Snf4p/Gal83p complex<br>307: Snf1p/Snf4p/Sip2p complex

311	312	311: Protein O-mannosyltransferase(Pmt3p/Pmt5p)<br>312: Protein O-mannosyltransferase(Pmt3p/Pmt5p)
313	460	313: SNARE complex<br>313: Sro1p/Sec4p/Sec9p complex<br>460: Sro1p/Sec4p/Sec9p complex
332	333	332: Psr1p/Whi2p complex<br>333: Psr1p/Whi2p complex
372	373	372: Sgv1p/Bur2p complex<br>373: Sgv1p/Bur2p complex
397	398	397: DNA polymerase delta complex<br>398: DNA polymerase delta complex
397	399	397: DNA polymerase delta complex<br>399: DNA polymerase delta complex
430	1296	430: GAL4p/GAL80p complex<br>430: GAL3p/GAL80p complex<br>1296: GAL3p/GAL80p complex
431	432	431: Synaptonemal complex (SC)<br>432: Synaptonemal complex (SC)
431	433	431: Synaptonemal complex (SC)<br>433: Synaptonemal complex (SC)
431	434	431: Synaptonemal complex (SC)<br>434: Synaptonemal complex (SC)
495	496	495: GET complex<br>496: GET complex
495	497	495: GET complex<br>497: GET complex
585	586	585: Rvs161p/Rvs167p complex<br>586: Rvs161p/Rvs167p complex
593	594	593: ARGR complex<br>594: ARGR complex
593	595	593: ARGR complex<br>595: ARGR complex
593	596	593: ARGR complex<br>596: ARGR complex
609	610	609: THO complex<br>609: Cdc73p/Paf1p complex<br>610: THO complex
609	611	609: THO complex<br>609: Cdc73p/Paf1p complex<br>611: THO complex
609	612	609: THO complex<br>609: Cdc73p/Paf1p complex<br>612: THO complex
613	614	613: cohesin loading factor complex<br>614: cohesin loading factor complex
616	1197	616: Pho85p/Pcl8p complex<br>616: Pho85p/Pho80p complex<br>616: Pho85p/Pcl10p complex<br>616: Pho85p/Pcl1p complex<br>616: Pho85p/Pcl2p complex<br>1197: Pho85p/Pcl10p complex
616	1575	616: Pho85p/Pcl8p complex<br>616: Pho85p/Pho80p complex<br>616: Pho85p/Pcl10p complex<br>616: Pho85p/Pcl1p complex<br>616: Pho85p/Pcl2p complex<br>1575: Pho85p/Pcl2p complex
682	683	682: adenylyl cyclase complex<br>683: adenylyl cyclase complex
691	692	691: Hse1p/Vps27p complex<br>692: Hse1p/Vps27p complex
715	716	715: Nsp1p/Nup82p complex<br>715: Nsp1p complex<br>716: Nsp1p/Nup82p complex<br>716: Nup82 nuclear pore subcomplex
717	718	717: Arg2p/Arg5,6p complex<br>718: Arg2p/Arg5,6p complex
754	1371	754: transcription factor TFIIIB complex<br>754: transcription factor TFIID complex<br>754: Mot1p complex<br>371: Mot1p complex
790	791	790: Cdc7p/Dbf4p complex<br>791: Cdc7p/Dbf4p complex
809	810	809: Gyl1p/Gyp5p complex<br>810: Gyl1p/Gyp5p complex
840	841	840: Bi3 ribonucleoprotein complex<br>841: Bi3 ribonucleoprotein complex
928	929	928: carbamoyl-phosphate synthase complex<br>929: carbamoyl-phosphate synthase complex
931	932	931: Bi4p/Nam2p complex
1084	1188	1084: Cdc24p/Far1p/Gbetagamma protein complex<br>1084: heterodimeric G-protein complex<br>1084: heterotrimeric G-protein complex<br>1188: heterotrimeric G-protein complex
1139	1140	1139: Sli15p/Bir1p complex<br>1140: Sli15p/Bir1p complex<br>1140: Aurora B-INCENP protein kinase complex
1140	1553	1140: Sli15p/Bir1p complex<br>1140: Aurora B-INCENP protein kinase complex<br>1553: Aurora B-INCENP protein kinase complex
1156	1157	1156: nucleotide-excision repair factor 1 complex<br>1157: nucleotide-excision repair factor 1 complex
1156	1158	1156: nucleotide-excision repair factor 1 complex<br>1158: nucleotide-excision repair factor 1 complex
1159	1160	1159: Tof1p/Mrc1p/Csm3p complex<br>1160: Tof1p/Mrc1p/Csm3p complex
1159	1161	1159: Tof1p/Mrc1p/Csm3p complex<br>1161: Tof1p/Mrc1p/Csm3p complex
1191	1192	1191: Clathrin<br>1192: Clathrin
1234	1235	1234: GSH degradosomal complex<br>1235: GSH degradosomal complex
1249	1560	1249: nucleotide-excision repair factor 2 complex<br>1249: Png1p/Rad23p complex
1271	1272	1271: Def1p/Rad26p complex<br>1272: Def1p/Rad26p complex
1283	1284	1283: Holliday junction resolvase complex<br>1284: Holliday junction resolvase complex
1328	1329	1328: LMA1 complex<br>1329: LMA1 complex
1339	1340	1339: translation release factor complex<br>1340: translation release factor complex
1431	1432	1431: glycosylphosphatidylinositol-N-acetylglucosaminyltransferase (GPI-GnT) complex<br>1432: glycosylphosphatidylinositol-N-acetylglucosaminyltransferase (GPI-GnT) complex
1431	1433	1431: glycosylphosphatidylinositol-N-acetylglucosaminyltransferase (GPI-GnT) complex<br>1433: glycosylphosphatidylinositol-N-acetylglucosaminyltransferase (GPI-GnT) complex
1458	1459	1458: 1,3-beta-glucan synthase complex (Gsc2p/Smk1p)<br>1459: 1,3-beta-glucan synthase complex (Gsc2p/Smk1p)
1484	1485	1484: Sod1p/Ccs1p complex<br>1485: Sod1p/Ccs1p complex
1521	1522	1521: signal recognition particle receptor complex<br>1522: signal recognition particle receptor complex
1529	1530	1529: anthranilate synthase complex<br>1530: anthranilate synthase complex
1615	1616	1615: Nem1p/Spo7p complex<br>1616: Nem1p/Spo7p complex

Table A15 : Frequency distribution of the assigned categories of additional proteins on iRefIndex from PANTHER analysis, (a) Biological process (b) Cellular component (c) Molecular function. FQ shows the count for each matched category.

(a) 74 categories of biological process.

Category of biological process	FQ
cellular process	47
nitrogen compound metabolic process	45
biosynthetic process	27
phosphate-containing compound metabolic process	16
response to stress	16
DNA repair	13
catabolic process	9
regulation of nucleobase-containing compound metabolic process	9
DNA replication	8
regulation of cell cycle	8
cell cycle	7
cytoskeleton organization	6
regulation of biological process	6
tRNA metabolic process	6
regulation of phosphate metabolic process	6
regulation of transcription from RNA polymerase II promoter	6
DNA recombination	5
organelle organization	5
mitosis	5
cellular amino acid biosynthetic process	5
organelle organization	5
reproduction	4
protein localization	4
response to stimulus	4
cellular protein modification process	4
cellular component organization	3
cellular amino acid metabolic process	3
cellular component biogenesis	3
regulation of carbohydrate metabolic process	3
chromosome segregation	3
RNA splicing, via transesterification reactions	3
tRNA aminoacylation for protein translation	3
nucleobase-containing compound transport	3
cellular component organization	3
nuclear transport	3
nucleobase-containing compound metabolic process	3
mRNA splicing, via spliceosome	3
RNA localization	3
intracellular signal transduction	3
meiosis	3
intracellular protein transport	2
G-protein coupled receptor signaling pathway	2
developmental process	2
single-multicellular organism process	2
cellular component morphogenesis	2
glycogen metabolic process	2
protein targeting	2
proteolysis	2
transcription elongation from RNA polymerase II promoter	2
DNA metabolic process	2
cytokinesis	2
chromatin organization	2
receptor-mediated endocytosis	2
cyclic nucleotide metabolic process	2
regulation of catalytic activity	2
exocytosis	1
RNA metabolic process	1
protein methylation	1
rRNA metabolic process	1
monosaccharide metaboli process	1
ion transport	1
vesicle-mediated transport	1
response to toxic substance	1
MAPK cascade	1
pyrimidine nucleobase metabolic process	1
mitochondrial translation	1
signal transduction	1
mRNA processing	1
protein phosphorylation	1
phagocytosis	1
response to abiotic stimulus	1
transcription initiation from RNA polymerase II promoter	1
transcription from RNA polymerase II promoter	1
phospholipid metabolic process	1

(b) 26 categories of cellular component.

Category of cellular component	FQ
intracellular	30
protein complex	30
nucleus	26
cytoplasm	25
nuclear chromosome	10
nucleoplasm	7
integral to membrane	5
plasma membrane	5
protein-DNA complex	4
endoplasmic reticulum	3
cytosol	3
ribonucleoprotein complex	3
Golgi apparatus	3
microtubule	3
extracellular space	2
mitochondrion	2
mitochondrial inner membrane	2
membrane	2
heterotrimeric G-protein complex	2
vesicle coat	2
nuclear envelope	2
nuclear outer membrane-endoplasmic reticulum membrane network	1
cell part	1
cytoplasmic membrane-bounded vesicle	1
actin cytoskeleton	1
microtubule organizing cente	1

(c) 56 categories of molecular function.

Category of molecular function	FQ
protein binding	12
protein kinase activity	9
GTPase activity	6
phosphoprotein phosphatase activity	6
DNA-directed DNA polymerase activity	6
binding	5
pyrophosphatase activity	5
nucleotidyltransferase activity	4
damaged DNA binding	4
DNA binding	4
chromatin binding	3
RNA binding	3
kinase activity	3
sequence-specific DNA binding transcription factor activity	3
hydrolase activity	3
aminoacyl-tRNA ligase activity	3
oxidoreductase activity	3
endodeoxyribonuclease activity	3
phosphatase regulator activity	3
nucleotide binding	3
sequence-specific DNA binding RNA polymerase II transcription factor activity	3
single-stranded DNA binding	2
structural constituent of cytoskeleton	2
antioxidant activity	2
kinase activator activity	2
transferase activity, transferring glycosyl groups	2
metallopeptidase activity	2
signal transducer activity	2
enzyme activator activity	2
adenylate cyclase activity	2
transferase activity	2
ligase activity	2
kinase regulator activity	1
actin binding	1
phosphatase activator activity	1
lipid binding	1
lyase activity	1
structural molecule activity	1
isomerase activity	1
acetyltransferase activity	1
translation release factor activity	1
translation factor activity, nucleic acid binding	1
nucleic acid binding	1
RNA methyltransferase activity	1
small GTPase regulator activity	1
ubiquitin-protein ligase activity	1
nuclease activity	1
transporter activity	1
guanyl-nucleotide exchange factor activity	1
carbohydrate kinase activity	1
nucleotide phosphatase activity	1
receptor binding	1
transmembrane transporter activity	1
exodeoxyribonuclease activity	1
mRNA binding	1
calcium ion binding	1

Table A16 : 67 additional protein pairs on BioGRID and S_{all} which is the estimated score by PSOPIA.

Name of gene encoding the protein		S_{all}
SBH2	SSH1	0.9810
SBH2	SSS1	0.3537
MSS1	MTO1	0.3613
INH1	STF2	0.3537
INH1	ATP19	0.3537
SMB1	PRP28	0.7761
HOP1	ZIP2	0.3537
IMG1	MRPL31	0.3537
IMG1	MRPL33	0.0000
IMG1	MRPL38	0.3537
IMG1	MRPL49	0.3537
DYN1	DYN3	0.3537
LPD1	GCV1	0.3537
LPD1	GCV2	0.8325
LPD1	GCV3	0.3537
ARG2	ARG5,6	0.3537
COB	COR1	0.3537
COB	QCR10	0.3537
COB	QCR7	0.3537
COB	QCR8	0.3537
COB	QCR9	0.0000
MET10	MET5	0.7617
RSE1	YSF3	0.3537
COX1	COX12	0.3537
COX1	COX13	0.0000
MGE1	PAM17	0.3537
DSS1	SUV3	0.5786
RPL10	RPL14A	0.3537
RPL10	RPL15B	0.3537
RPL10	RPL21B	0.3537
RPL10	RPL22A	0.3537
RPL10	RPL22B	0.3537
RPL10	RPL34A	0.3537
RPL10	RPL34B	0.3537
RPL10	RPL37A	0.3537
RPL10	RPL37B	0.3537
RPL10	RPL39	0.3537
RPL10	RPL40A	0.3613
RPL10	RPL40B	0.3613
RPL10	RPL41C	0.0000
RPL10	RPL41B	0.0000
RPL10	RPL42A	0.3537
RPL10	RPS43A	0.3537
SDH1	SDH3	0.3537
PBI2	TRX1	0.3537
ASC1	RPS10A	0.8731
ASC1	RPS12	0.4523
ASC1	RPS16A	0.3497
ASC1	RPS19A	0.3497
ASC1	RPS21A	0.1820
ASC1	RPS21B	0.1820
ASC1	RPS23B	0.5563
ASC1	RPS26A	0.3461
ASC1	RPS27A	0.3197
ASC1	RPS27B	0.3197
ASC1	RPS28A	0.0000
ASC1	RPS29A	0.3537
ASC1	RPS29B	0.4187
ASC1	RPS30B	0.2851
ASC1	RPS31	0.2058
ERI1	GPI1	0.3537
ERI1	GPI15	0.3537
ERI1	GPI19	0.3537
TOM20	TOM6	0.3537
HSC82	SRO9	0.8966
MRP1	PPE1	0.3537
MRP1	YMR31	0.3537

Table A17: Comparison of prediction performance with the four databases using unweighted datasets. A 'PIPE' data is an original PPI data predicted from PIPE method. A 'Combined' data is generated by combing the PPIs from the corresponding existing method with those on GreedyMinPPI.

(a) Using STRING

Method	Recall (%)	Precision (%)	Specificity (%)	ACC (%)	F-measure (%)	MCC
GreedyMinPPI	0.102	79.4	100.0	94.9	0.203	0.0273
PIPE	0.984	68.2	100.0	95.0	1.94	0.0779
Combined	1.04	68.7	100.0	95.0	2.05	0.0805
SPPS	1.48	63.6	100.0	95.0	2.90	0.0918
Combined	1.54	64.0	100.0	95.0	3.01	0.0938
InteroPORC	0.00	0.00	99.9	96.1	–	-0.00162
Combined	0.153	53.6	100.0	94.9	0.305	0.0266

(b) Using MINT

Method	Recall (%)	Precision (%)	Specificity (%)	ACC (%)	F-measure (%)	MCC
GreedyMinPPI	2.90	19.8	100.0	99.2	5.06	0.0734
PIPE	14.4	8.71	99.9	99.8	10.8	0.111
Combined	15.3	8.80	99.9	99.8	11.2	0.115
SPPS	15.6	5.86	99.8	99.8	8.50	0.0947
Combined	16.5	5.98	99.8	99.8	8.78	0.0982
InteroPORC	0.011	0.060	99.8	98.7	1.80	-0.00453
Combined	2.91	8.91	99.9	99.5	4.39	0.0488

(c) Using WI-PHI

Method	Recall (%)	Precision (%)	Specificity (%)	ACC (%)	F-measure (%)	MCC
GreedyMinPPI	2.03	75.4	100.0	99.7	4.00	0.123
PIPE	18.3	60.4	100.0	99.7	28.1	0.331
Combined	19.2	60.4	100.0	99.7	29.2	0.340
SPPS	19.9	40.7	99.9	99.7	26.7	0.283
Combined	20.8	41.2	99.9	99.7	27.6	0.291
InteroPORC	0.00	0.00	100.0	99.8	–	-0.00039
Combined	2.15	35.9	100.0	99.7	4.06	0.0874

(d) Using IntAct

Method	Recall (%)	Precision (%)	Specificity (%)	ACC (%)	F-measure (%)	MCC
GreedyMinPPI	10.2	35.6	99.9	99.6	15.8	0.189
PIPE	70.9	22.0	99.9	99.9	33.6	0.394
Combined	72.6	21.4	99.9	99.9	33.1	0.394
SPPS	73.3	14.1	99.8	99.8	23.6	0.321
Combined	74.7	13.9	99.8	99.8	23.4	0.321
InteroPORC	0.00	0.00	99.9	99.8	–	-0.00108
Combined	10.2	15.6	99.9	99.7	12.4	0.126

$$Recall = \frac{TP}{TP+FN}, \qquad Precision = \frac{TP}{TP+FP},$$
$$Specificity = \frac{TN}{TN+FP}, \qquad ACC = \frac{TP+TN}{TP+FP+FN+TN},$$
$$F\text{-}measure = \frac{2TP}{(TP+FP)+(TP+FN)},$$
$$MCC = \frac{(TP \times TN)-(FP \times FN)}{\sqrt{(TP+FP)\times(TP+FN)\times(TN+FP)\times(TN+FN)}},$$

based on $TP(TruePositives)$, $FP(FalsePositives)$, $TN(TrueNegatives)$ and $FN(FalseNegatives)$.

Table A18: Comparison of prediction performance using AUC with STRING.

	Struct2Net	ENTS	PIP	iWRAP
Original	0.642	0.612	0.561	-
Combined (all)				
SPE = 0.1	0.657	0.629	0.572	0.931
SPE = 1.0	0.662	0.635	0.580	0.994
SPE = 5.0	0.663	0.635	0.581	0.999
SPE = 10.0	0.663	0.636	0.581	0.999
Combined (200)				
SPE = 0.1	0.582	0.588	0.540	0.453
SPE = 1.0	0.582	0.588	0.540	0.453
SPE = 5.0	0.582	0.588	0.540	0.453
SPE = 10.0	0.616	0.588	0.540	0.453
Combined (500)				
SPE = 0.1	0.629	0.602	0.552	0.494
SPE = 1.0	0.629	0.602	0.553	0.494
SPE = 5.0	0.629	0.602	0.553	0.494
SPE = 10.0	0.629	0.602	0.553	0.494

Table A19: Comparison of prediction performance using AUC with MINT.

	Struct2Net	ENTS	PIP	iWRAP
Original	0.684	0.715	0.565	0.515
Combined (all)				
SPE = 0.1	0.762	0.753	0.634	0.928
SPE = 1.0	0.801	0.772	0.754	0.989
SPE = 5.0	0.801	0.772	0.763	0.989
SPE = 10.0	0.801	0.772	0.764	0.989
Combined (200)				
SPE = 0.1	0.582	0.678	0.441	0.282
SPE = 1.0	0.582	0.678	0.442	0.282
SPE = 5.0	0.582	0.678	0.442	0.282
SPE = 10.0	0.582	0.678	0.442	0.282
Combined (500)				
SPE = 0.1	0.641	0.702	0.531	0.495
SPE = 1.0	0.641	0.702	0.540	0.495
SPE = 5.0	0.641	0.702	0.541	0.495
SPE = 10.0	0.641	0.702	0.541	0.495

Table A20: Comparison of prediction performance using AUC with WI-PHI.

	Struct2Net	ENTS	PIP	iWRAP
Original	0.702	0.704	0.558	0.555
Combined (all)				
SPE = 0.1	0.768	0.735	0.626	0.848
SPE = 1.0	0.793	0.747	0.699	0.908
SPE = 5.0	0.796	0.749	0.709	0.913
SPE = 10.0	0.735	0.749	0.711	0.914
Combined (200)				
SPE = 0.1	0.573	0.648	0.428	0.242
SPE = 1.0	0.573	0.648	0.429	0.242
SPE = 5.0	0.573	0.648	0.429	0.242
SPE = 10.0	0.573	0.648	0.429	0.242
Combined (500)				
SPE = 0.1	0.631	0.675	0.499	0.422
SPE = 1.0	0.631	0.675	0.504	0.422
SPE = 5.0	0.631	0.675	0.504	0.422
SPE = 10.0	0.631	0.675	0.504	0.422

Table A21: Comparison of prediction performance using AUC with IntAct.

	Struct2Net	ENTS	PIP	iWRAP
Original	0.671	0.703	0.581	0.616
Combined (all)				
SPE = 0.1	0.795	0.748	0.695	0.932
SPE = 1.0	0.813	0.759	0.800	0.985
SPE = 5.0	0.816	0.759	0.811	0.991
SPE = 10.0	0.816	0.758	0.812	0.991
Combined (200)				
SPE = 0.1	0.542	0.660	0.406	0.253
SPE = 1.0	0.542	0.660	0.408	0.253
SPE = 5.0	0.542	0.660	0.408	0.253
SPE = 10.0	0.542	0.660	0.408	0.253
Combined (500)				
SPE = 0.1	0.631	0.694	0.527	0.493
SPE = 1.0	0.631	0.694	0.538	0.493
SPE = 5.0	0.631	0.694	0.538	0.493
SPE = 10.0	0.631	0.694	0.538	0.493

References

[1] Fields S, Song O. A novel genetic system to detect protein-protein interactions. Nature. 1989 Jul 20;340(6230):245–246. PMID:2547163.

[2] Rigaut G, Shevchenko A, Rutz B, Wilm M, Mann M, Sraphin B. A genetic protein purification method for protein complex characterization and proteome exploration. Nature biotechnology. 1999 Oct;17(10):1030–1032. PMID:16278461.

[3] Collins SR, Kemmeren P, Zhao XC, Greenblatt JF, Spencer F, Holstege FC. Toward a comprehensive atlas of the physical interactome of Saccharomyces cerevisiae. Bioinformatics. 2007 Mar;6(3):439–450. PMID:17456562.
